# Differences and similarities between the genetic architecture of lifetime substance use across different substances

**DOI:** 10.1017/S0033291725101293

**Published:** 2025-07-30

**Authors:** Uri Bright, Cassie Overstreet, Daniel F. Levey, Joel Gelernter

**Affiliations:** 1 Department of Psychiatry, Yale School of Medicine, New Haven, CT, USA; 2 Department of Psychiatry, Veterans Affairs Connecticut Healthcare System, West Haven, CT, USA; 3 Departments of Genetics and Neuroscience, Yale School of Medicine, New Haven, CT, USA

**Keywords:** genomewide analyses, GWAS, psychiatric traits, substance use

## Abstract

**Background:**

Illicit drug use may lead to dependence on those drugs, is associated with various psychiatric disorders, and can have hazardous, sometimes life-threatening, consequences. We investigated the genetic architecture underlying the lifetime use (LU) of several drugs, individually and in combination.

**Methods:**

We conducted genome-wide association studies of LU of cocaine, methamphetamine, inhalants, illegal opioids, prescription opioids, and prescription stimulants in European (EUR), African (AFR), and Latin American (AMR)-ancestry subjects (cases ranging from *n* = 4,900–21,850 [EUR], *n* = 519–9,802 [AFR], and *n* = 899–5,012 [AMR]; controls from *n* = 93,763–110,658 [EUR], *n* = 37,261–46,509 [AFR], and *n* = 31,412–35,501 [AMR]). We also investigated the use of illicit drugs of any kind and the total count of drugs a person has ever used. Then, we assessed the global and local genetic correlations between substance LU (SubLU) traits and their genetic correlations with other traits.

**Results:**

We found numerous genes that affect SubLU traits, with no overlap among the significant loci between traits, suggesting that unique genetic factors may differentially affect the use of different drugs. Nevertheless, the genetic correlations between SubLU traits were very strong; however, the phenotypic correlations were moderate. There were strong genetic correlations between various SubLU traits and psychiatric traits, most notably opioid use disorder, cannabis use disorder, problematic alcohol use, and suicidality.

**Conclusions:**

Our findings provide insights into the genetic basis of substance use, identifying several novel genes associated with SubLU traits. This study can provide an improved understanding of the biology underlying SubLU and could potentially facilitate future risk assessments for the use of illicit and hazardous drugs.

## Background

Illicit and hazardous drugs, such as cocaine, methamphetamine, inhalants, stimulants, and opioids, pose significant health risks, contributing to injuries, psychiatric and physical diseases, and an increased risk of death (Afonso, Mohammad, & Thatai, 2007; Isoardi et al., 2020; Jones & Rayner, 2015; Kaye, McKetin, Duflou, & Darke, 2007; Maraj, Figueredo, & Morris, 2010; Mick, McManus, & Goldberg, 2013; Valente et al., 2012; van der Woude, 2000; Verna, Schluger, & Brown, 2019). Nevertheless, drug use is a widespread phenomenon with increasing prevalence (Barocas et al., 2018; Han et al., 2021; John & Wu, 2017; Wu & Ringwalt, 2006; Yockey, King, & Vidourek, 2020). Therefore, both environmental and genetic factors influencing the use of these drugs are major public health concerns.

In 2023, the past-year prevalence of cocaine use in the United States was 1.8%, inhalant use was 0.9%, and methamphetamine use was 0.9%. In addition, there was a past-year 1.4% frequency of prescription stimulant use and 3.1% use of legal and illegal opioids (SAMHSA, 2023). The lifetime prevalence of opioid use and misuse was between 11.9% and 37.8% (Han et al., 2017; Zajacova et al., 2023), while the prevalence of illegal street opioid use, such as heroin, was between 1.6% and 1.84% (Ihongbe & Masho, 2016; Martins et al., 2017). The lifetime prevalence of prescription stimulant use is 9.5% (McCabe & West, 2013).

Transition rates from initial use to developing a use disorder or addiction vary between different drugs. For example, between 0.3% and 0.4% of the population reportedly develops cocaine use disorder (CocUD) (John & Wu, 2017; Kerridge et al., 2019) (suggesting a transition rate of up to ~2.5%), whereas 0.8–4.6% develop opioid use disorder (OUD) (Barocas et al., 2018; Han et al., 2017) (suggesting a transition rate of up to ~39%). There is also high comorbidity between the use of different types of drugs; for example, cocaine use is prevalent in 11.8% of heavy alcohol users, with 2.10% of this population developing CocUD (John & Wu, 2017). In addition, 73% of individuals with OUD report co-use of other substances (Mahoney, Marshalek, Haut, Hodder, et al., 2021), and 27.5% of them develop alcohol use disorder (Baskerville et al., 2023). Tobacco smoking is strongly associated with opioid use and OUD (Rajabi et al., 2019).

There is also high comorbidity between substance use and various psychiatric disorders. The point prevalence of depression is 24% among cocaine-dependent users (López & Becoña, 2007) and 27–61% among opioid users (Rogers et al., 2021), compared to 5% in the general population (WHO, 2023). The odds of depression among methamphetamine users are 18–66% higher than in the general population (Leung et al., 2023) and 32% higher among inhalant users (Gentile, Bianco, Nordstrom & Nordstrom, 2021). Other disorders, such as attention-deficit/hyperactivity disorder (ADHD) (Obermeit et al., 2013; Oliva et al., 2021; Wilens et al., 2008), schizophrenia (Hunt et al., 2018), post-traumatic stress disorder (PTSD) (Dahlby & Kerr, 2020), and bipolar disorder (BD) (Cerullo & Strakowski, 2007), are also more common in people with histories of substance use. Drugs, such as cocaine, opioids, and stimulants, pose health risks other than psychiatric effects, which can deeply impair the quality of life and may be life-threatening; these include hypertension, elevated heart rate, myocardial infarction and heart failure (Afonso et al., 2007; Kaye et al., 2007; Maraj et al., 2010; Mick et al., 2013), liver damage (Valente et al., 2012; Verna et al., 2019; Zhao et al., 2020), and kidney injury (Isoardi et al., 2020; Jones & Rayner, 2015; Valente et al., 2012; van der Woude, 2000). Out of 600,000 drug-related deaths in a 37-year period, the vast majority were caused by cocaine, prescription opioids, and street opioid overdose (Jalal et al., 2018). However, this study did not include alcohol- and tobacco-related deaths, which are estimated to be even higher (Karaye, Maleki, Hassan, & Yunusa, 2023; Siegel et al., 2015). Opioid abuse has been considered a national epidemic in the United States (Volkow & Blanco, 2021), with annual opioid-associated mortality rates rising to 73,838 in 2022 (NIH, 2022). In total, ~150,000 people die every year in the United States due to drug overdose (CDC, 2024).

Most genome-wide association studies (GWASs) of substance use traits have dealt with substance use frequency, substance dependence, and substance use disorders (SUDs) (Dao et al., 2021; Deak et al., 2022; Levey et al., 2023; Sanchez-Roige et al., 2019; Zhou et al., 2023), and less so with substance lifetime use (LU) (with the exception of tobacco smoking initiation, which is similar to an LU trait; e.g. see Saunders et al., 2022). Although it is clear why SUDs are of great interest, the liability to use drugs should be investigated too, considering the high transition rates from use to use disorder and possible hazards that arise from nondependent use. In addition, studies conducted so far regarding cannabis (Levey et al., 2023; Pasman et al., 2018) and alcohol (Saunders et al., 2022; Zhou et al., 2023), for example, indicate that substance LU (SubLU) and SUD traits are somewhat different from one another genetically. Here, we conducted GWAS analyses to investigate the genetic basis of LU of cocaine, methamphetamine, inhalants, illegal opioids, prescription opioids, and prescription stimulants in European (EUR), African (AFR), and Latin American (AMR) ancestry research participants. We examined the genetic and phenotypic similarities and differences between these traits and their global and local genetic correlations with traits of interest, including chronic pain, SUDs, depression, and other psychiatric, medical, and socioeconomic traits. We also evaluated the genetic underpinnings of the inclination to use illicit drugs of any kind, as well as the amount of drugs a person uses over a lifetime.

## Methods

See also Supplementary Methods.

### Cohorts

We included subjects of EUR, AFR, and AMR ancestries from the All of Us (AoU; v7) biobank. Genotyping and quality control procedures were described previously (Bick et al., 2024). LU of cocaine, methamphetamine, inhalants, street opioids, prescription opioids, and prescription stimulants phenotypes were defined using a lifestyle survey. The ‘any substance’ (SubLU) and quantitative ‘number of substances used’ (nSubLU) phenotypes were defined using the same survey.

### GWAS analysis and meta-analysis

GWAS analyses were conducted using PLINK 2.0, with sex, age, and the first 10 genetic PCs as covariates. We removed subjects due to relatedness, and variants were excluded due to minor allele frequency <0.1% and Hardy–Weinberg equilibrium *p* < 1 × 10^−6^. Cross-ancestry meta-analyses were performed using METAL (Willer, Li, & Abecasis, 2010).

### Genetic correlations and SNP-based heritability

We used linkage disequilibrium score regression (LDSC) (Bulik-Sullivan et al., 2015) to calculate single-nucleotide polymorphism (SNP)-based heritability (*h* ^2^) for all the traits and inter-trait genetic correlations between all six individual SubLU traits. Then, we calculated the genetic correlation between each trait, including the composite traits SubLU and nSubLU, and 12 selected traits (Deak et al., 2022; Demontis et al., 2023; Docherty et al., 2023; Johnston et al., 2019; Levey et al., 2021; Levey et al., 2023; Nievergelt et al., 2024; O’Connell et al., 2025; Trubetskoy et al., 2022; Watanabe et al., 2022; Zhou et al., 2023) (Supplementary Table S1). For this analysis, we selected substance use traits with well-powered GWAS available. Psychiatric traits were included if they were previously phenotypically associated with substance use traits, such as schizophrenia, depression, BD, and PTSD (Dahlby & Kerr, 2020; Hunt et al., 2018; López & Becoña, 2007; Rogers et al., 2021), or if they are commonly associated with the use of, or treatment via, specific substances (e.g. ADHD, which is associated with stimulant use [Wilens et al., 2008], and chronic pain, which is associated with opioid use [Weiss et al., 2014]). The trait ‘academic degree’ was selected to represent cognitive function (Braatveit, Torsheim, & Hove, 2018).

### Phenotypic correlations

We estimated inter-trait phenotypic correlations using *χ* ^2^- and *φ*-coefficient (*r*_φ_).

### Local genetic correlations

We used local analysis of covariant association (LAVA) (Werme, van der Sluis, Posthuma, & de Leeuw, 2022) to calculate inter-trait local genetic correlations between all six SubLU traits (a total of 15 pairs). Then, we calculated the local genetic correlations between each trait – including SubLU and nSubLU – and 12 selected traits of interest (Supplementary Table S1).

### Cross-ancestry genetic correlations

We used Popcorn (Brown et al., 2016) to calculate the cross-ancestry genetic correlations between the SubLU trait in AFR and AMR populations and a selected list of traits in EUR. We used the same set of traits that were measured for genetic correlation among EUR using LDSC (see above). We applied the Benjamini–Hochberg procedure for correction of false discovery rate (FDR).

### Genomic structural equation modeling (gSEM)

We utilized gSEM (Grotzinger et al., 2019) to examine the underlying latent factor structure of the six individual SubLU traits, along with nine other psychiatric and health-related traits associated with substance use specifically.

## Results

### GWAS analyses

The average number of participants for the analysis of the six SubLU traits (cocaine, methamphetamine, inhalants, street opioids, prescription opioids, and prescription stimulants) was 115,578 in EUR, 47,039 in AFR, and 36,407 in AMR (for details, see Table 1). Demographics for the nSubLU trait are presented in Supplementary Table S2. For each trait, we also conducted a cross-ancestry meta-analysis. All lead SNPs were significant in the range of 5 × 10^−8^ and 1 × 10^−9^, except as noted (Table 2). Fifteen analyses yielded at least one significant SNP (Manhattan plots and regional plots in Supplementary Figures S1–15).

**Table 1.** Sample size for each trait

Substance (LU)	EUR		AFR		AMR	
	Case	Control	Case	Control	Case	Control
Cocaine	21,850	93,763	9,802	37,261	5,012	31,412
Methamphetamine	10,739	104,840	1,242	45,786	2,126	34,279
Prescription stimulants	13,536	102,039	2,295	44,746	2,245	34,159
Inhalants	6,788	108,775	519	46,509	899	35,501
Street opioids	4,900	110,658	2,383	44,651	1,207	35,194
Prescription opioids	12,074	103,504	2,786	44,257	2,291	34,118
All substances	34,017	81,618	12,904	34,182	7,316	29,123

**Table 2.** Lead SNPs of substance lifetime use traits

Substance (LU)	Ancestry	rsID	Ch*r*	Pos (h38)	p	Gene	Protein
Cocaine	AFR	rs78664860	4	186548657	3.88E−08	*MTNR1A*	Melatonin receptor 1A
	AMR	–					
	EUR	rs146999751	3	85444443	4.43E−08	*CADM2*	Cell adhesion molecule 2
		rs60331671	10	102250539	1.98E−08	*GBF1*	Golgi brefeldin A-resistant guanine nucleotide exchange factor 1
	Meta-analysis	rs9869718	3	82064905	3.20E−08	*LINC02008*	Long intergenic nonprotein-coding RNA 2008
		rs4856591	3	85563400	2.28E−08	*CADM2*	Cell adhesion molecule 2
		rs78664860	4	186548657	3.88E−08	*MTNR1A*	melatonin receptor 1A
		rs72930769	18	55491419	3.90E−08	*TCF4*	transcription factor 4
Inhalants	AFR	–					
	AMR	rs141550494	8	52895329	3.25E−08	–	–
		rs11699278	20	54172253	2.31E−08	*CYP24A1*	Cytochrome P450 family 24 subfamily A member 1
	EUR	rs4235547	5	22195653	1.87E−08	*CDH12*	Caherin–12
		rs77469549	19	40635573	2.05E−08	–	–
	Meta-analysis	rs112202767	1	40964121	1.83E−08	–	–
		rs72637502	8	52881639	3.39E−08	–	–
Methamphetamine	AFR	–					
	AMR	rs141493660	8	78235188	8.58E−10	–	–
	EUR	–					
	Meta-analysis	rs17431748	8	118078108	2.91E−08	*EXT1*	Exostosin glycosyltransferase 1
Street opioids	AFR	rs113325386	3	65465078	2.01E−08	*MAGI1*	Membrane-associated guanylate kinase, WW and PDZ domain-containing 1
	AMR	–					
	EUR	–					
	Meta-analysis	rs113325386	3	65465078	2.01E−08	*MAGI1*	Membrane-associated guanylate kinase, WW and PDZ domain-containing 1
Prescription opioids	AFR	rs141109612	19	34977173	2.81E−08	–	–
	AMR	rs149549558	11	71589856	1.64E−08	–	–
		rs72989824	11	99423774	1.74E−08	*CNTN5*	Contactin 5
	EUR	–					
	Meta-analysis	rs1052457445	19	35026372	1.04E−08	*GRAMD1A*	GRAM domain containing 1A
Prescription stimulants	AFR	–					
	AMR	rs55775765	17	82759850	4.45E−08	*TBCD*	Tubulin folding cofactor D
	EUR	–					
	Meta-analysis	rs116758901	5	88702511	3.98E−08	*MEF2C-AS2* ^a^	
Any substance (SubLU)	AFR	rs1600561869	19	53618688	5.32E−09	*DPRX*	Civergent-paired related homeobox
	AMR	–					
	EUR	rs1821351	3	85439175	1.29E−10	*CADM2*	cell adhesion molecule 2
		rs7931884	11	28626831	1.55E−10	*LINC02758*	Long intergenic nonprotein-coding RNA 2758
		rs4813097	20	1359305	2.75E−08	*SDCBP2-AS1; FKBP1A-SDCBP2*	SDCBP2 Antisense RNA 1; FKBP1A-SDCBP2
	Meta-analysis	rs76518228	1	232289714	9.08E−09	–	
		rs1821351	3	85439175	7.09E−10	*CADM2*	Cell adhesion molecule 2
		rs12489967	3	170355551	2.91E−08	–	–
		rs116350745	5	159009642	4.93E−08	*EBF1*	Early B-cell factor 1
		rs7931884	11	28626831	3.93E−09	*LINC02758*	long intergenic nonprotein-coding RNA 2758
Number of substances (0–6) (nSubLU)	AFR	rs141793374	18	10563190	4.69E−08	–	
	AMR	rs17465728	4	80232838	1.10E−08	*LOC124900725* ^a^	
		rs149549558	11	71589856	1.48E−09	–	
	EUR	rs60331671	10	102250539	6.32E−09	*GBF1*	Golgi brefeldin A-resistant guanine nucleotide exchange factor 1
		rs7931884	11	28626831	1.14E−08	*LINC02758*	Long intergenic nonprotein coding RNA 2758
	Meta-analysis	rs2275399	1	236718103	2.31E−08	*ACTN2*	Actinin alpha 2
		rs35149938	2	59939243	3.15E−08	–	
		rs327133	3	108088638	2.52E−08	*CD47*	CD47
		rs1594670302	13	96084351	3.33E−08	–	
		rs141793374	18	10563190	4.69E−08	–	
		rs28758902	18	55740956	1.79E−08	*LOC105372130* ^a^	

a Noncoding RNA.

For Cocaine LU, we found two genome-wide significant (GWS) variants in EUR (CADM2*rs146999751 and GBF1*rs60331671) and one in AFR (MTNR1A*rs78664860). A cross-ancestry meta-analysis revealed four significant loci: LINC02008*rs9869718, CADM2*rs4856591, TCF4*rs72930769, and MTNR1A*rs78664860. For Inhalants LU, we found two significant hits: CDH12*rs4235547 in EUR and CYP24A1*rs11699278 in AMR. A cross-ancestry meta-analysis revealed two intergenic hits. For Methamphetamine LU, we found one significant variant in AMR in a noncoding region (rs141493660, *p* = 8.6 × 10^−10^) and a different one in a cross-ancestry meta-analysis (EXT1*rs17431748). For Street Opioids LU, we found a significant hit only in AFR (MAGI1*rs113325386), which was preserved in the cross-ancestry meta-analysis. For Prescription Opioids LU, we found one significant protein-coding locus in AMR (CNTN5*rs7298982), and one in a cross-ancestry meta-analysis (GRAMD1A*rs1052457445). For Prescription Stimulants LU, we found significant hits in AMR (TBCD*rs55775765), and in a cross-ancestry meta-analysis (MEF2C-AS2*rs116758901).

We then conducted a GWAS of composite (cumulative) traits: an ‘any substance’ LU (SubLU) trait, in which cases were defined as subjects that used any of the substances included for the analyses described above; and a quantitative trait of ‘number of substances used’ (nSubLU), defined as the number of substances subjects listed as ‘ever used’ in their lifetime (a range of 0–6 substances). The number of participants used for the analysis was 115,635 in EUR, 47,086 in AFR, and 36,439 in AMR (Supplementary Table S2). All lead SNPs were significant in the range of 5 × 10^−8^ and 10^−9^, unless stated otherwise (Table 2).

**Figure 1.**
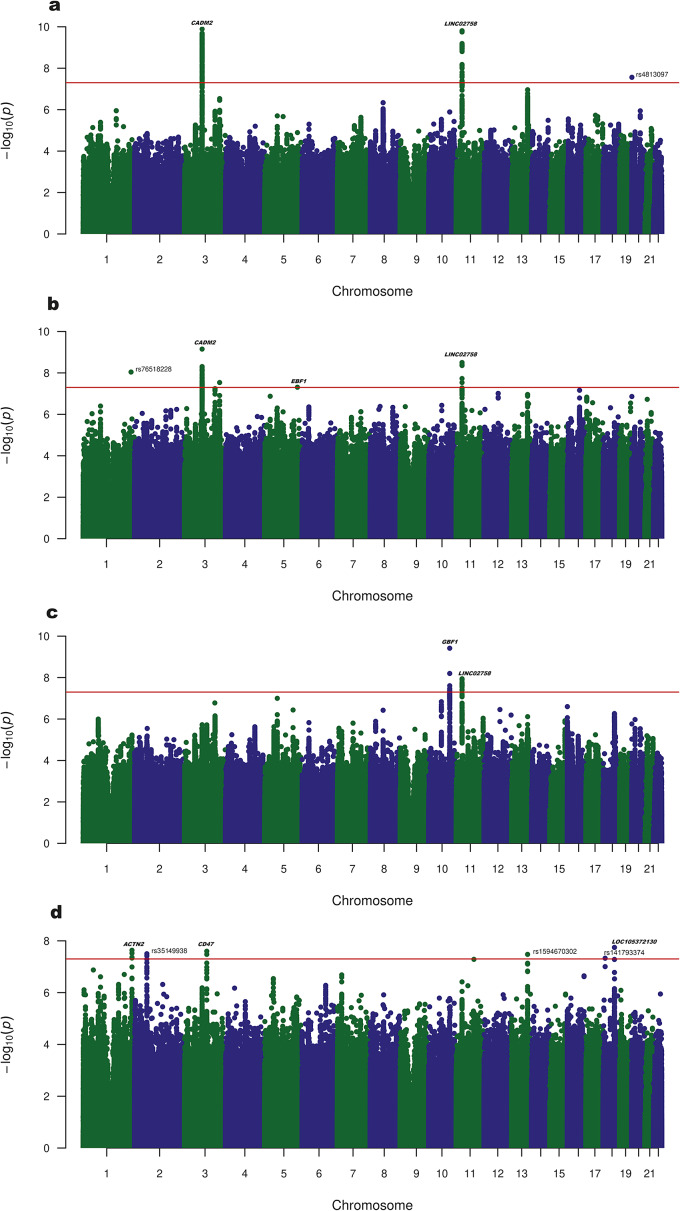
Manhattan plot of substance lifetime use (LU) (LU of one or more of the drugs discussed in this study) in (a) EUR (nCases = 34,017, nControls = 81,618); (b) cross-ancestry meta-analysis (nCases = 54,237, nConrols = 144,923), and number of lifetime substances used (the number of different drugs, of the drugs discussed in this study, and a subject used in his or her lifetime; a quantitative trait with a range of 0–6) in (c) EUR (*n* = 115,635); (d) cross-ancestry meta-analysis (*n* = 199,190).

For SubLU, the ‘any substance’ trait, we found three significant variants in EUR (CADM2*rs1821351, *p* = 1.29 × 10^−10^; LINC02758*rs7931884, *p* = 1.55 × 10^−10^; and rs4813097, which is located in an overlapping area of two genes: *SDCBP2-AS1* and *FKBP1A-SDCBP2*) (Figure 1a) and one in AFR (DPRX*rs1600561869) (Supplementary Figure S16). A cross-ancestry meta-analysis revealed two significant loci that were also significant for EUR (CADM2*rs1821351, *p* = 7.09 × 10^−10^, and LINC02758*rs7931884) and three new significant SNPs (EBF1*rs116350745 and the intergenic rs76518228 and rs12489967) (Figure 1b and Supplementary Figure S19–20).

For nSubLU, the quantitative trait, we found two hits in EUR (GBF1*rs60331671 and LINC02758*rs7931884) (Figure 1c and Supplementary Figure S21): one in AFR (the intergenic rs141793374) (Supplementary Figure S17), and two in AMR (LOC124900725*rs17465728 and the intergenic rs149549558) (Supplementary Figure S18). A cross-ancestry meta-analysis revealed six SNPs that were significantly associated with nSubLU: the AFR-bound rs141793374 and five that were not significant for the individual ancestries: ACTN2*rs2275399, CD47*rs327133, LOC105372130*rs28758902, and the intergenic rs35149938 and rs58899690 (Figure 1d and Supplementary Figure S22).

### Inter-trait genetic correlations

**Figure 2.**
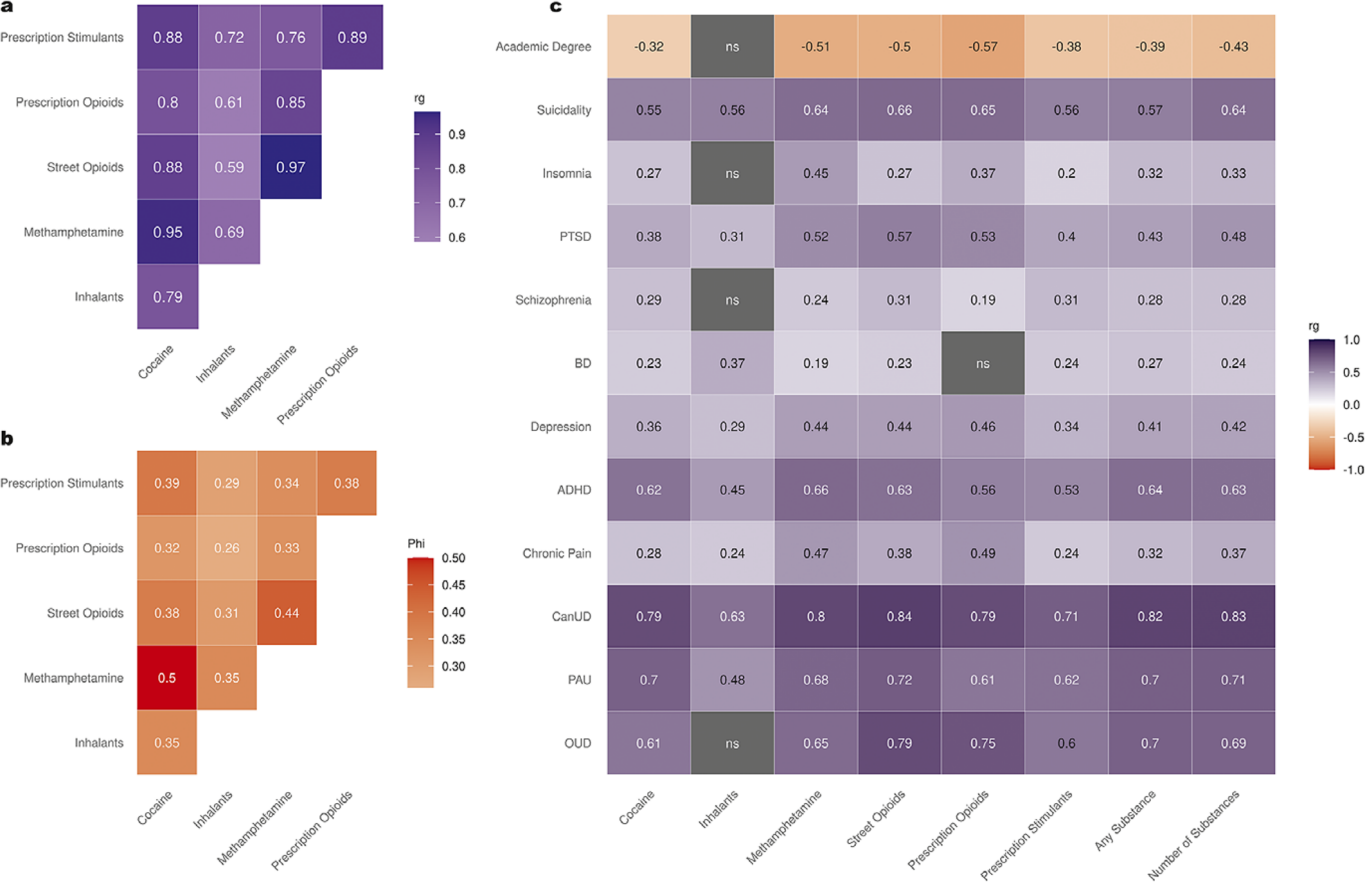
(a) Inter-trait genetic correlations between all six individual substance lifetime use (LU) traits in EUR. (b) Inter-trait phenotypic correlations between all six individual substance LU traits in EUR. (c) Genetic correlations between all six individual and cumulative substance LU traits in EUR and a selected list of traits. Statistically nonsignificant values are in dark gray Note: ADHD, ‘attention-deficit/hyperactivity disorder’; BD, ‘bipolar disorder’; CanUD, ‘cannabis use disorder’; ns, ‘nonsignificant’; OUD, ‘opioid use disorder’; PAU, ‘problematic alcohol use’; PTSD, ‘post-traumatic stress disorder’ [Deak et al., 2022; Demontis et al., 2023; Doherty et al., 2018; Johnston et al., 2019; Levey et al., 2023; Levey et al., 2021; Nievergelt et al., 2024; O’Connell et al., 2025; Trubetskoy et al., 2022; Watanabe et al., 2022; Zhou et al., 2023].

LDSC was used to calculate SNP-based heritability (*h* ^2^) and inter-trait genetic correlations for the individual SubLU traits in EUR. Heritability estimates ranged between 8.08% (for Street Opioids LU; SE = 0.009) and 13.9% (for Prescription Opioids LU; SE = 0.019). Intercept measures ranged between 0.992 and 1.012, and attenuation ratios were of 0.085 or lower (Supplementary Table S3). Inter-trait genetic correlations were calculated between all six individual SubLU traits – a total of 15 pairs. A significant positive moderate-to-strong correlation was found between all pairs. The strongest correlations were between Street Opioids LU and Prescription Opioids LU (*r*_g_ = 0.968, *p* = 5.87 × 10^−68^), Street Opioids LU and Methamphetamine LU (*r*_g_ = 0.965, *p* = 9.96 × 10^−129^), and Cocaine LU and Methamphetamine LU (*r*_g_ = 0.95, *p* = 1.66 × 10^−133^). In total, 12 pairs of traits had a strong genetic correlation of *r*_g_ > 0.7 (Figure 2a and Supplementary Table S4).

### Inter-trait phenotypic correlations

Inter-trait phenotypic correlations were calculated for individual SubLU traits using *χ* ^2^-estimates and *φ*-coefficients. The analyses were conducted separately for EUR, AFR, and AMR, and revealed positive weak-to-moderate phenotypic correlations between all the traits in all the ancestries (Supplementary Table S5). In EUR (Figure 2b) and AMR (Supplementary Figure S23), the strongest correlation was observed between Cocaine LU and Methamphetamine LU (*r*_φ_ = 0.5 and *r*_φ_ = 0.47, respectively). In AFR (Supplementary Figure S24), the strongest correlation was between Cocaine LU and Street Opioids LU (*r*_φ_ = 0.31).

### Inter-trait local genetic correlations

Inter-trait local genetic correlations were calculated for individual SubLU traits in EUR using LAVA (Werme et al., 2022). For every trait, only regions that reached the significance threshold of *p* < 0.05 were used to calculate genetic correlations with the other traits (a total of 3,242 regions in 15 pairs). After Bonferroni correction for 3,242 tests, the statistically significant threshold was set on *p* = 1.54 × 10^−5^. All 15 pairs had genetic correlations in at least three regions, and all the correlations were positive. The most highly correlated traits were Street Opioids LU and Methamphetamine LU (40 significantly correlated genetic regions), Street Opioids LU and Prescription Opioids LU (37 regions), and Street Opioids LU and Inhalants LU (37 regions). The most prominent region was region 1,225 (chr7:152253295–153241228), which genetically correlated eight pairs (the full data are presented in Supplementary Table S6).

### Correlations between SubLU traits and other traits of interest

LDSC was used to calculate genetic correlations between the individual SubLU traits in EUR and 12 selected traits related to substance use, substance dependence, and common psychiatric illnesses (Figure 2c and Supplementary Table S7). In total, 72 pairs were analyzed (6 substance use traits × 12 ‘other’ traits); there were only 4 instances of nonsignificant (but nominally correlated) relationships, all of them involving Inhalants LU. For all other pairs, there were significant genetic correlations with a wide spectrum of statistical significance: the weakest effect was for Prescription Opioids LU and schizophrenia (*r*_g_ = 0.193, *p* = 4.64 × 10^−6^) and the strongest was for Street Opioids LU and cannabis use disorder (CanUD) (*r*_g_ = 0.843, *p* = 1.82 × 10^−66^). For 10 of the 11 traits tested, all the correlations with SubLU traits were positive, while for academic degree, all the correlations were negative. The two strongest correlations of OUD were with opioid use traits (Street Opioids LU: *r*_g_ = 0.789, *p* = 2.4 × 10^−24^; Prescription Opioids LU: *r*_g_ = 0.749, *p* = 4.31 × 10^−14^), and the strongest correlation of chronic pain was with Prescription Opioids LU (*r*_g_ = 0.487, *p* = 4.05 × 10^−25^). Even though prescription stimulants are prescribed mostly to treat ADHD (Piper et al., 2018), Methamphetamine LU (*r*_g_ = 0.665, *p* = 5.10 × 10^−45^) and Cocaine LU (*r*_g_ = 0.6212, *p* = 8.10 × 10^−43^) had higher genetic correlation with ADHD compared to Prescription Stimulants LU (*r*_g_ = 0.528, *p* = 4.02 × 10^−26^). We also calculated the genetic correlations between SubLU and nSubLU and the same 12 selected traits. All the tests had significant results. For all the traits, the *r*_g_ values with SubLU and nSubLU were highly similar. Nevertheless, in all cases, the correlation with nSubLU had much lower *p*-values.

### Local genetic correlations between SubLU traits and other traits of interest

Local genetic correlations between individual SubLU traits in EUR and other traits of interest were calculated using LAVA (Werme et al., 2022). For every trait, only regions that reached the significance threshold of *p* < 0.05 were used to calculate genetic correlations with the other traits (a total of 15,841 regions in 96 pairs). After Bonferroni correction for 15,841 tests, the statistical significance threshold was set at *p* = 3.16 × 10^−6^. In total, 25 significant correlations between individual and cumulative SubLU traits and the other traits of interest were found. Methamphetamine LU was the SubLU trait with the greatest number of shared regions, with six different associations, two of them with CanUD. One region – 1,292 (chr8: 55275355–56346878) – was associated with problematic alcohol use (PAU) as well as two individual LU traits: Inhalants LU and Methamphetamine LU. Region 1,966 (chr14:33591114–34695195) associated schizophrenia with both Prescription Stimulants LU and nSubLU. Region 727 (chr4:139553761–141087047) associated chronic pain with Prescription Stimulants LU and BD with Street Opioids. Region 267 (chr2:59251997–60775066) associated CanUD with Methamphetamine LU, BD with Cocaine LU, academic degree with SubLU, and BD with SubLU. All the other significant regions were associated with one pair of traits (Supplementary Table S8).

### Cross-ancestry genetic correlations

Using Popcorn (Brown et al., 2016), genetic correlations were calculated for SubLU in AMR and AFR against a set of traits of interest in EUR. After FDR correction, there were three significant correlations in AFR – for chronic pain, suicidality, and academic degree – and eight in AMR – for OUD, PAU, CanUD, chronic pain, ADHD, depression, BD, and suicidality. In all the cases, the correlation with SubLU was in the same direction as in EUR (as calculated by LDSC) (Supplementary Table S9 and Supplementary Figure S25).

### gSEM modeling

**Figure 3.**
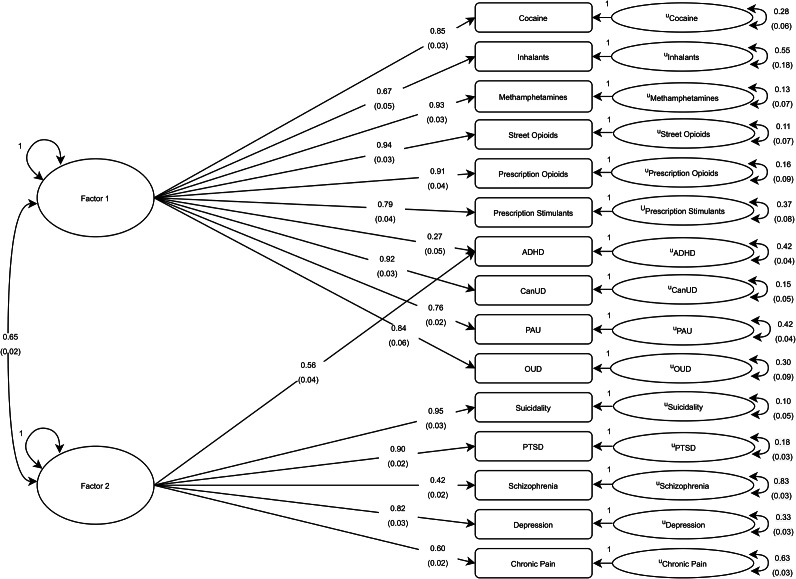
Path diagram for genomic structural equation modeling for confirmatory factor analysis (CFA) results of the two-factor model. The diagram presents the results of the correlated two-factor CFA model of 15 substance use, psychiatric, and chronic pain traits for European ancestry participants. Standardized estimates are provided for each path with standard errors included in parentheses.

Heritability estimates and genetic correlations were calculated across traits using LDSC (Supplementary Table S10). Parallel analysis indicated that a two-factor model best fit the data, although a third factor was relatively close to reaching the threshold for consideration (Supplementary Figure S26). Therefore, we performed two exploratory factor analyses (two- and three-factor solutions). Comparisons across the two- and three-factor model EFA results suggested that the two-factor model best fit the data due to the minimal amount of variance accounted for by inclusion of the third factor (<0.06). For the two-factor EFA, cocaine, inhalants, methamphetamines, street opioids, prescription opioids, prescription stimulants, CanUD, PAU, and OUD (i.e. substance-related traits) loaded onto factor 1, while suicidality, PTSD, schizophrenia, depression, and chronic pain (i.e. nonsubstance psychiatric traits) loaded onto factor 2. ADHD cross-loaded onto both factors. A confirmatory factor analysis (CFA) was performed, inputting traits on the relevant factor as indicated by EFA loading results, and the CFA results indicated adequate fit with comparative fit index (CFI) = 0.9476 and standardized root mean square residual (SRMR) = 0.0784 (Figure 3 and Supplementary Table S11).

## Discussion

Substance use and use disorders, even for the same substance, have often been seen to differ genetically (Levey et al., 2023; Pasman et al., 2019; Sanchez-Roige et al., 2019; Zhou et al., 2020) and, therefore, need to be studied individually. While there have been numerous studies of alcohol and tobacco use traits (Buchwald et al., 2021; Sanchez-Roige et al., 2019; Xu et al., 2020; Zhou et al., 2023) and several for cannabis (Levey et al., 2023; Pasman et al., 2018), many substance use traits have not yet been subject to genetic study. We present here the first genetic investigations for several of these traits. We found several genes associated with different SubLU traits, with no overlap among the significant loci between traits: although there were very high genetic correlations between these traits (in EUR), different lead SNPs – located within different genes – were significantly associated with each of these phenotypes. This may, however, reflect power limitations, that is, as more cases become available for each substance and more loci are identified, it is plausible that more loci common to multiple traits may be identified.

For the individual SubLU traits, there were a total of three hits in AFR, two of them within protein-coding genes. For Cocaine LU, the *MTNR1A* gene had a significant effect (all significant SNPs mentioned in this section had a *p*-value in the range of 5 × 10^−8^–1 × 10^−10^). This gene encodes one of the two main melatonin receptors, known for its major role in circadian rhythms and the sleep–wake cycle (Hardeland, Pandi-Perumal, & Cardinali, 2006). There is a strong association between sleep disorders and SUDs (Conroy & Arnedt, 2014), and specifically cocaine (Schierenbeck, Riemann, Berger, & Hornyak, 2008), and melatonin was used as an experimental treatment for SUDs, with mixed results (Das, Prithviraj, & Mohanraj, 2022). The effect of *MTNR1A* on Cocaine LU may provide a window into the interaction between sleep disorders and cocaine use, especially considering that it was also significant in a cross-ancestry meta-analysis. For Street Opioids LU, there was a hit in an intronic variant of *MAGI1.* Its encoded protein participates in inter-cell interactions and has been associated with neuroticism (Genetics of Personality et al., 2015), BD, and schizophrenia (Karlsson et al., 2012).

To our knowledge, there have been no previous genetic studies of inhalant use or dependence. In AMR, rs11699278 – an intronic variant of *CYP24A1*, a gene involved in the regulation of vitamin D and calcium homeostasis (Jones, Prosser, & Kaufmann, 2012) – was associated with Inhalants LU; it did not have high LD with any significant (or nearly significant) SNPs (Supplementary Figure S4), so this finding might be a false-positive. There was a significant effect of *CNTN5*, a member of the contactin gene family, on Prescription Opioids LU. This gene has a role in cell-surface interactions during the developmental phase of the nervous system. Variants are associated with Alzheimer’s disease (Dauar et al., 2023), autism (van Daalen et al., 2011), and ADHD (Lionel et al., 2011).

In EUR, four loci were associated with individual SubLU traits. *GBF1* had a significant effect on Cocaine LU. The protein encoded by this gene has a role in diseases that affect muscular function, such as Charcot–Marie–Tooth (Mendoza-Ferreira et al., 2020) and Parkinson’s disease (PD) (Zhao et al., 2020). The latter might suggest its involvement in dopaminergic processes: PD is related to dopaminergic cell death (Latif et al., 2021), while substance use elevates dopamine release in the synapses (Trifilieff & Martinez, 2014). Cocaine acts via dopamine transporter inhibition (Gether, Andersen, Larsson, & Schousboe, 2006). In a multi-trait analysis of GWAS, *GBF1* had a significant effect on CanUD (Xu et al., 2023). *CADM2* was also associated with Cocaine LU in EUR, as well as cross-ancestry, in line with numerous studies suggesting that *CADM2* is involved in substance use traits and risk-taking behavior (Arends et al., 2021; Koller et al., 2024; Pasman et al., 2022; Sanchez-Roige et al., 2023; Zhou et al., 2020). For Inhalants LU, there was a lead SNP located in the *CDH12* gene, encoding a protein that was associated with infertility and endometriosis in females (Golawski et al., 2022). It has also been previously associated with schizophrenia (Hawi et al., 2018).

Cross-ancestry meta-analyses revealed additional novel associations: *TCF4* had a significant effect on Cocaine LU; this gene is associated with Pitt–Hopkins syndrome, a disorder of severe developmental delay and intellectual disability (Sweatt, 2013), and also with PTSD reexperiencing (Gelernter et al., 2019), corneal endothelial dystrophy, schizophrenia (Forrest et al., 2014), and depression (Mossakowska-Wojcik et al., 2018). *LINC02008*, a gene associated with blood pressure (Chen et al., 2021), affected Cocaine LU too. For Methamphetamine LU, there was one GWS variant that maps to *EXT1.* Variants at this locus are associated with exostoses (Ludecke et al., 1997) and were previously associated with other substance use traits such as smoking initiation (Saunders et al., 2022). As for Prescription Opioids LU, there was a cross-ancestry effect for a variant located within the *GRAMD1A* gene, encoding a protein responsible for cholesterol transfer. Studies link cholesterol levels to substance use (Buydens-Branchey & Branchey, 2003; Lin et al., 2012), suggesting that low plasma cholesterol may be associated with increased craving for drugs (Lin et al., 2012). For Prescription Stimulants, we found an effect for the noncoding RNA *MEF2C-AS2*, previously associated with sleep duration, physical activity (Doherty et al., 2018), and educational attainment (Okbay et al., 2022).

Genes that were significantly associated with the individual SubLU traits cover a broad range of functionality, yet the inter-trait genetic correlations were very high. Looking at the local inter-trait genetic correlations, two of the three most highly correlated pairs (Street Opioids LU–Methamphetamine LU and Street Opioids LU–Prescription Opioids LU, with 40 and 37 correlated regions, respectively) also had the highest *r*_g_ between them. The pair with the lowest observed *r*_g_, that is, Inhalants LU–Street Opioids LU, also had 37 common regions, an indication of the complexity of genetic correlations and of the hidden information that might be exposed using LAVA. In comparison, the phenotypic correlations between the traits were low to moderate, suggesting that the differences between the traits (the inclination to prefer one substance over another) may be based mostly on environmental factors. In other words, genetically, SubLU traits are quite similar to one another, yet factors such as substance availability (Kiang, Basu, Chen, & Alexander, 2019), prescription patterns (Schieber et al., 2019), income, socioeconomic status (Han et al., 2021; John & Wu, 2017; Patrick, Wightman, Schoeni, & Schulenberg, 2012; Skoog et al., 2014; Wu & Ringwalt, 2006), and education (Han et al., 2021; Skoog et al., 2014; Wu & Ringwalt, 2006) might lead people to choose one drug over the other.

We also calculated the genetic correlations between individual SubLU traits and 12 traits of interest. Generally, the prominent results came from the two opioid LU traits. As expected, Prescription and Street Opioids LU had the highest genetic correlations with OUD. Prescription Opioids LU had the highest genetic correlation with depression, which was less expected; a clinical study showed that the comorbidity of depression with OUD is actually lower than with CocUD or methamphetamine use disorder (MetUD) (Calarco & Lobo, 2021). The fact that the present findings with the same substances differ may reflect the differences between substance use and use disorders, as well as differences between genetic and phenotypic relationships. While initiation of cocaine use is mainly recreational (van der Poel et al., 2009), opioids are usually prescribed to treat pain (Weiss et al., 2014, which occurs in high comorbidity with depression (IsHak et al., 2018). Therefore, the fact that Prescription Opioids LU also had the highest genetic correlation with chronic pain was expected. Prescription Opioids LU also had the strongest negative correlation with having an academic degree, in accordance with findings regarding impairing effects of opioids on academic performance (Darolia, Owens, & Tyler, 2022; Ellis, Kasper, & Cicero, 2020). Moderate negative correlations between academic degree and methamphetamine and cocaine LU are also backed by literature (Dean, Morales, Hellemann, & London, 2018; Jeynes, 2022). A negative genetic correlation between Prescription Stimulants LU and academic degree is in line with its positive correlation with ADHD. The relatively low (compared to other pairs) correlation between Prescription Stimulants LU and ADHD, even though stimulants are mainly prescribed to treat the latter, was surprising. Generally, the trait that was most strongly associated with SubLU traits was CanUD, with a mean *r*_g_ of 0.76 (±0.073) among individual traits and >0.82 for the cumulative SubLU traits. This can be partially explained by the fact that cannabis is the most commonly used intoxicating drug in daily or near-daily use in the United States, even more than alcohol (5.21%) (Caulkins, 2024). Yet, daily or near-daily tobacco smoking was more common, with 11.6% prevalence (2022).

Given the high correlations indicating moderate to strong phenotypic and genetic associations across the substance use and psychiatric traits, we utilized gSEM to examine the genetic architecture encompassed by these phenotypes. This investigation revealed two latent genetic factors with substance use traits loading more strongly onto factor 1 and other traits (psychiatric disorders and chronic pain) loading strongly onto factor 2, with the exception of ADHD, which adequately cross-loaded onto both factors. These results align with existing data regarding common genetic architecture between different SUDs (Abdellaoui et al., 2021; Deak & Johnson, 2021; Hatoum et al., 2023) and are similar to previous findings that showed that substance use traits tend to align under the same latent factor, while other psychiatric traits usually fit under a separate psychopathology-oriented factor (Abdellaoui et al., 2021; Levey et al., 2023).

We conducted two further GWAS analyses of composite traits: (i) counting any substance use trait (of the substances included in this study) as an indicator of any-SubLU; and (ii) considering substance use as a quantitative trait (nSubLU), measuring the total number of substances used by each subject (a range of 0–6 substances), which can be viewed as a severity measure. By this, we examined whether the genetic factors that are associated with substance use in general are different, to a degree, from those that influence the severity of substance use, or the tendency to use multiple drugs. We found different loci associated with each of these traits, suggesting that different factors affect any-substance use and substance use severity as judged by the comorbidity of the same substances. In AFR, the only hit for nSubLU was in a noncoding region, while for SubLU, there was a significant variant within the *DPRX* gene, encoding a DNA-binding protein thought to be involved in embryonic development (Madissoon et al., 2016). In AMR, there were two GWS findings for nSubLU, though one has the appearance of a false positive, and the second maps to a noncoding RNA (Supplementary Figure S18).

In EUR, there was one mutual finding between SubLU and nSubLU, a variant that maps to *LINC02758*, which was previously associated with smoking initiation (Saunders et al., 2022), suicidal thoughts (Kimbrel et al., 2023), depression (Zhang et al., 2024), ADHD (Chen et al., 2024), and risk-taking behavior (Baselmans et al., 2022). For SubLU, its significance (*p* = 1.55 × 10^−10^) was higher compared to nSubLU (*p* = 1.14 × 10^−8^) by nearly two orders of magnitude, and only in SubLU it was significant for the cross-ancestry meta-analysis.

*CADM2* was significantly associated with SubLU in EUR and cross-ancestry, but not with nSubLU. As already mentioned, *CADM2* is one of the most strongly associated genes with substance use traits (Arends et al., 2021; Koller et al., 2024; Pasman et al., 2022; Sanchez-Roige et al., 2023; Zhou et al., 2020). These results support that it could be more important in the inclination to use substances, but less so for severity. We can also suggest the opposite regarding *GBF1*, which was not significantly associated with SubLU, but was with nSubLU, suggesting its possible involvement in increased risk taking. As with other differential associations, this could also reflect power differences between analyses and random variation. This effect of *GBF1* remained only in EUR, and did not appear cross-ancestry.

There was a significant association between nSubLU and *ACTN2*, previously associated with heart failure (Arvanitis et al., 2020), smoking initiation (Saunders et al., 2022), externalizing behavior (Karlsson Linner et al., 2024), and educational attainment (Okbay et al., 2022). *CD47*, associated with smoking initiation (Saunders et al., 2022) and BD (Li et al., 2021), was GWS for nSubLU too.

The genetic correlations of SubLU and nSubLU with traits of interest were almost identical. Nevertheless, some differences in local genetic correlation were revealed: nSubLU was locally correlated with schizophrenia and PAU (the latter association in a region near *CADM2*, suggesting a possible mediation of this association by *CADM2*), which showed more severe traits that suggest a greater association between nSubLU and pathologies. In Cross-ancestry, we found similar genetic correlations between SubLU and chronic pain across all three ancestries. In AMR, SubLU also had a significant genetic correlation with OUD, PAU, CanUD, ADHD, and depression, while in AFR, there was a genetic correlation between SubLU and academic degree. Nevertheless, these correlations were not as strikingly similar to those found in EUR, which may reflect the lower power in AFR analyses compared to EUR. These findings indicate that a similar underlying genomic architecture drives the genetic correlations between SubLU and chronic pain across different ancestries, whereas other traits exhibit greater dissimilarities.

To our knowledge, this is the first study to examine the genetic mechanisms behind a broad range of SubLU traits. Therefore, nearly all the results presented here are novel. We found genes that suggest a possible association between SubLU and some neurological disorders, including AD, PD, and autism. We also pointed to a genetic association with psychiatric phenomena like psychosis, depression, ADHD, and SUDs, observed through several mutual genes, such as *CADM2*, *TCF4*, *LINC02758*, and *CD47*, and via genetic correlations. The genetic correlations between SubLU traits were very high, but no gene had a significant effect on more than one individual trait. Most findings in the cumulative traits were novel too (i.e. did not appear in the individual substances). Our findings offer insights into the genetic predisposition to substance use. This knowledge could facilitate future risk assessments for substance use and provide improved understanding of the biology that underlies these traits.

This study has limitations. First, all the data used was based on the AoU cohort. AoU is a high-quality US sample with good representation from a range of US populations, although it is still important to explore these traits in other cohorts to confirm our results and be able to generalize them to other populations. Better powered studies might also reveal mutual (pleiotropic) effects of specific SNPs or loci between SubLU traits, which we were not able to detect in the current study due to limited sample sizes. Future availability of well-powered datasets for a variety of additional phenotypically associated traits like CocUD and MetUD may provide important insights regarding a broader range of genetic correlations between these phenotypes and SubLU traits. Second, all substance lifetime users were considered as a singular group, with no differentiation between occasional and high-frequency users, including people with SUD. Third, substance use traits were defined by self-report; this could impair the nature of assigning participants to the right group (i.e. case or control), considering the possible inaccuracies that may appear in a self-reported trait, and especially considering the sensitivity of reporting the use of an illegal substance. Fourth, for some of the traits, the sample sizes for the AFR and AMR populations were relatively small and, therefore, lacked the power to detect an effect that might exist. Future studies with larger sample sizes for those ancestries may reveal significant effects that did not appear in the current study.

## Supporting information

Bright et al. supplementary material Bright et al. supplementary material

## Supplementary material

The supplementary material for this article can be found at http://doi.org/10.1017/S0033291725101293.
