# Supplementary material for: Differences and similarities between the genetic architecture of lifetime substance use across different substances
Source: Psychol Med. 2025 Jul 30;55:e219. doi: 10.1017/S0033291725101293 (PMC12341034; doi:10.1017/S0033291725101293)
Supplement: Bright et al. supplementary material [file S0033291725101293sup001.zip › S0033291725101293sup001/Supplementary Figures.docx]

**
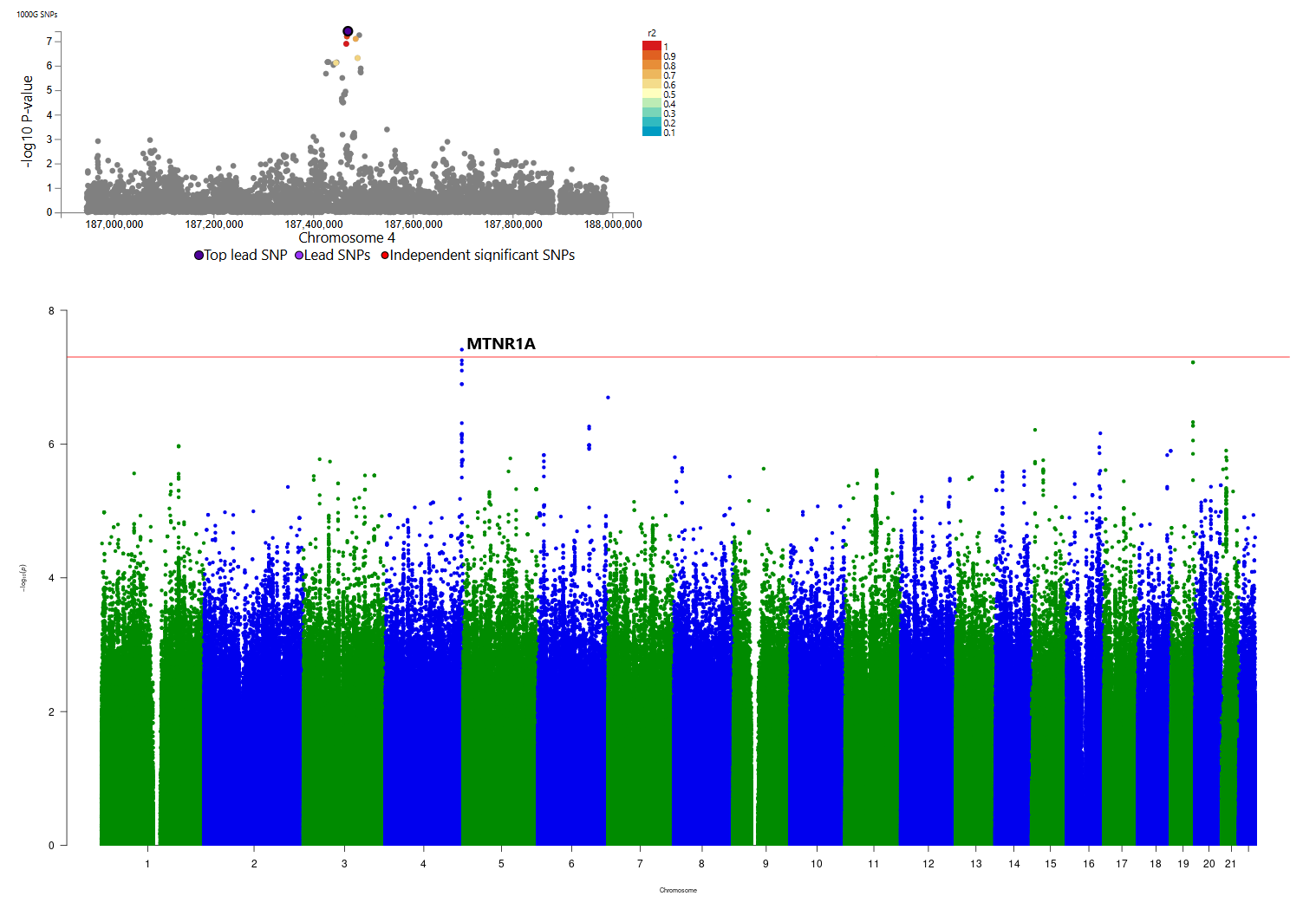
Figure S1:** Manhattan plot and regional plots of Cocaine Lifetime Use in AFR

**
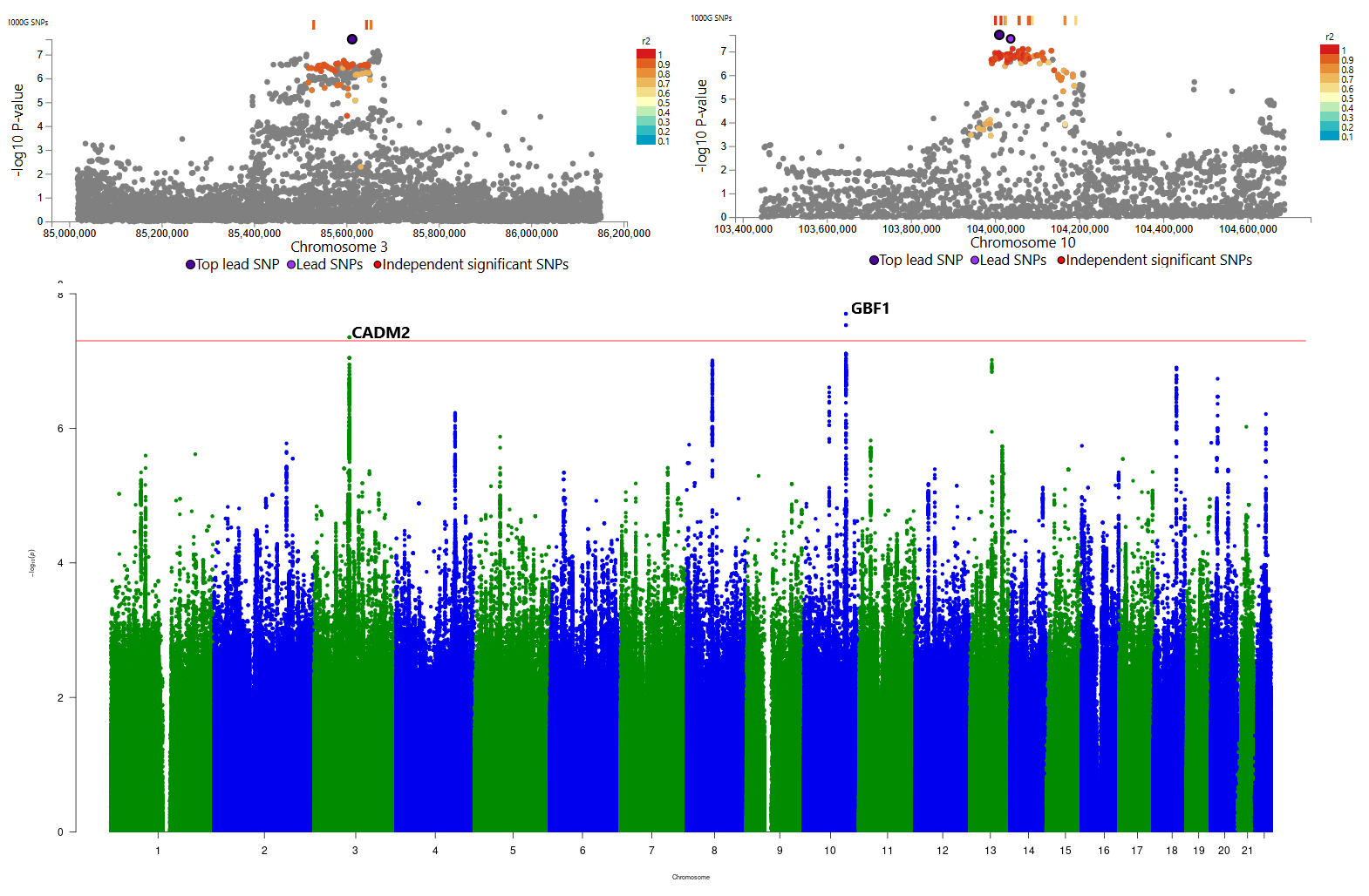
Figure S2:** Manhattan plot and regional plots of Cocaine Lifetime Use in EUR

**
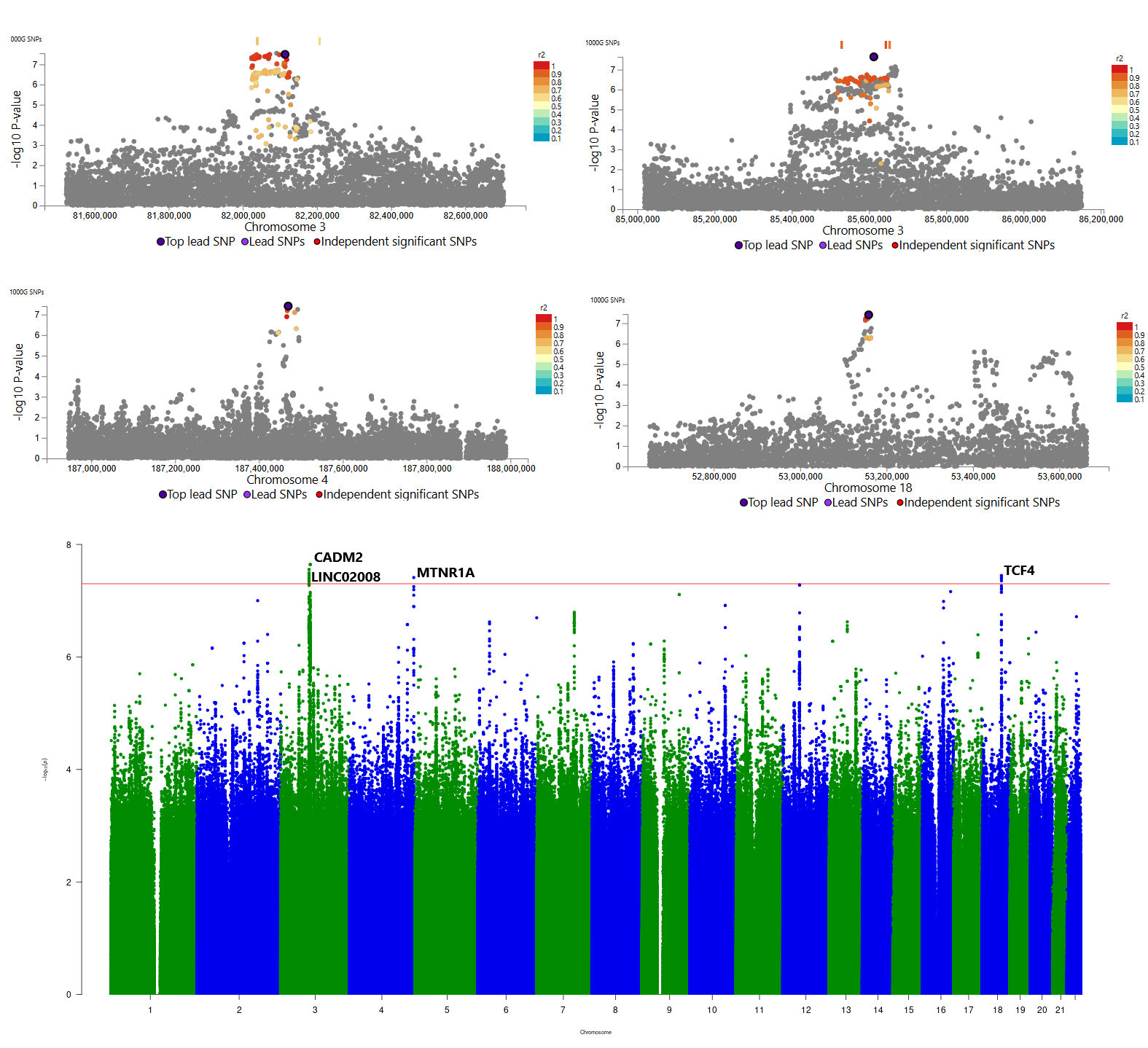
Figure S3:** Manhattan plot and regional plots of Cocaine Lifetime Use in a cross-ancestry meta-analysis

**
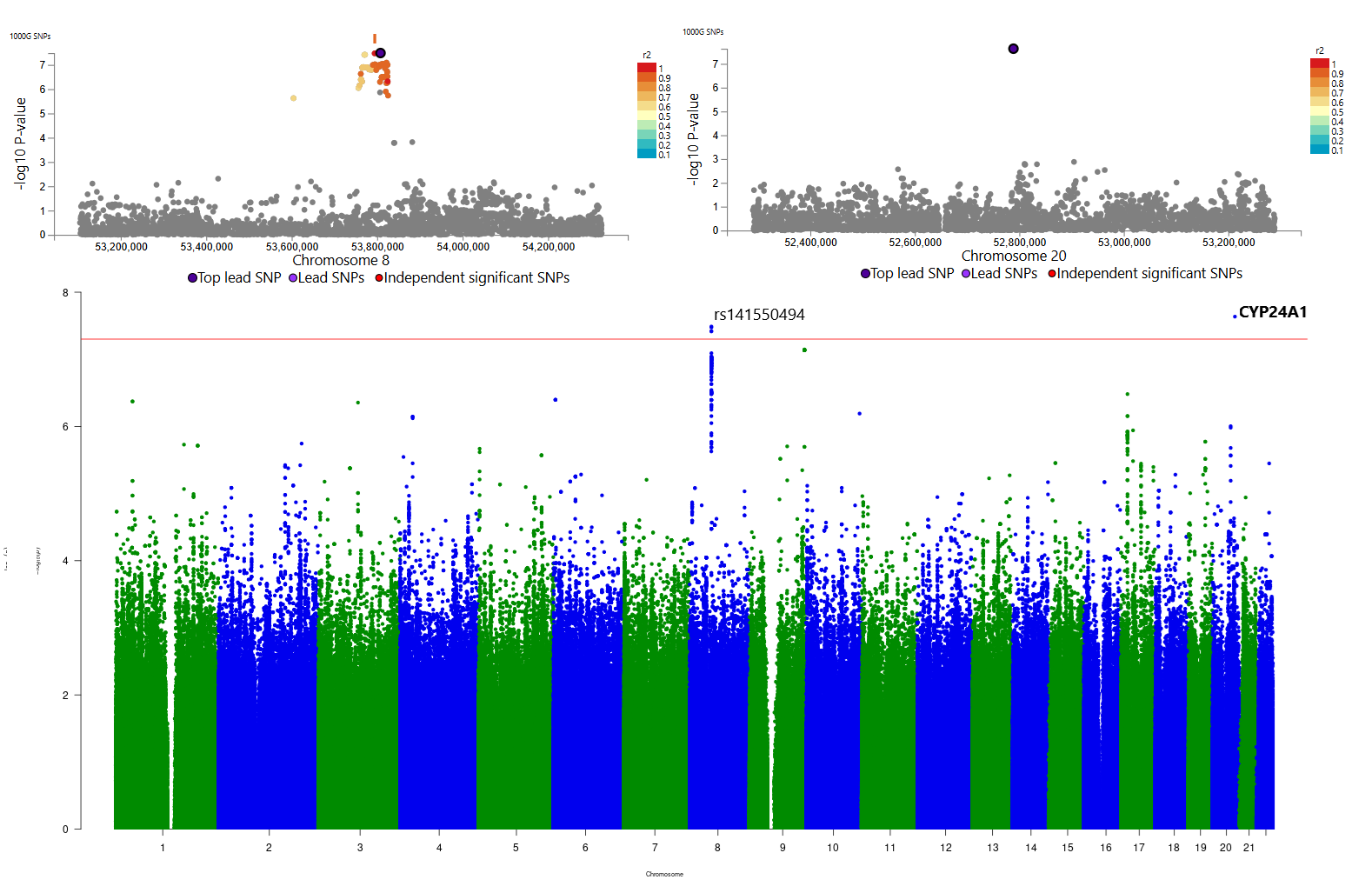
Figure S4:** Manhattan plot and regional plots of Inhalants Lifetime Use in AMR

**
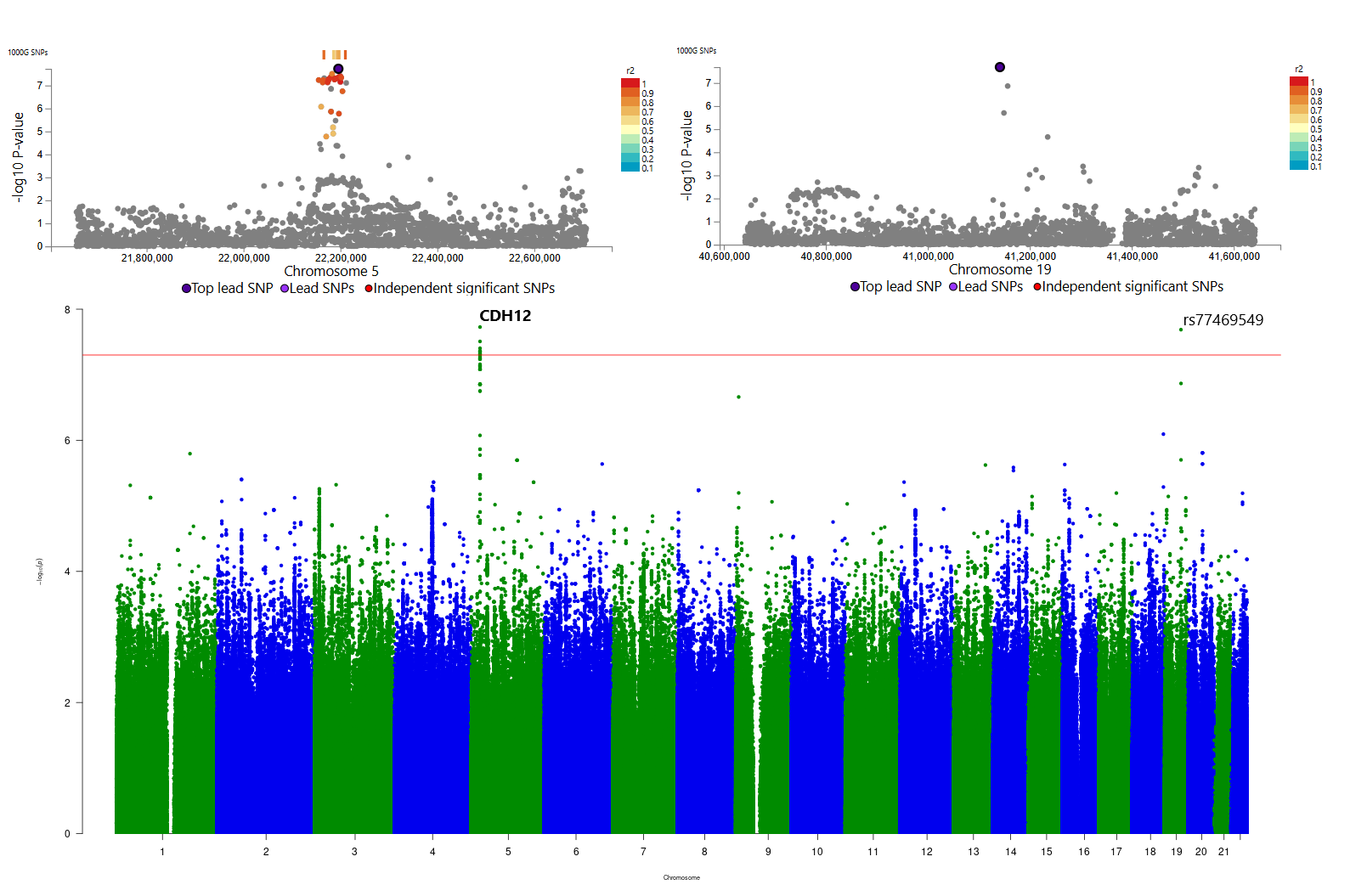
Figure S5:** Manhattan plot and regional plots of Inhalants Lifetime Use in EUR


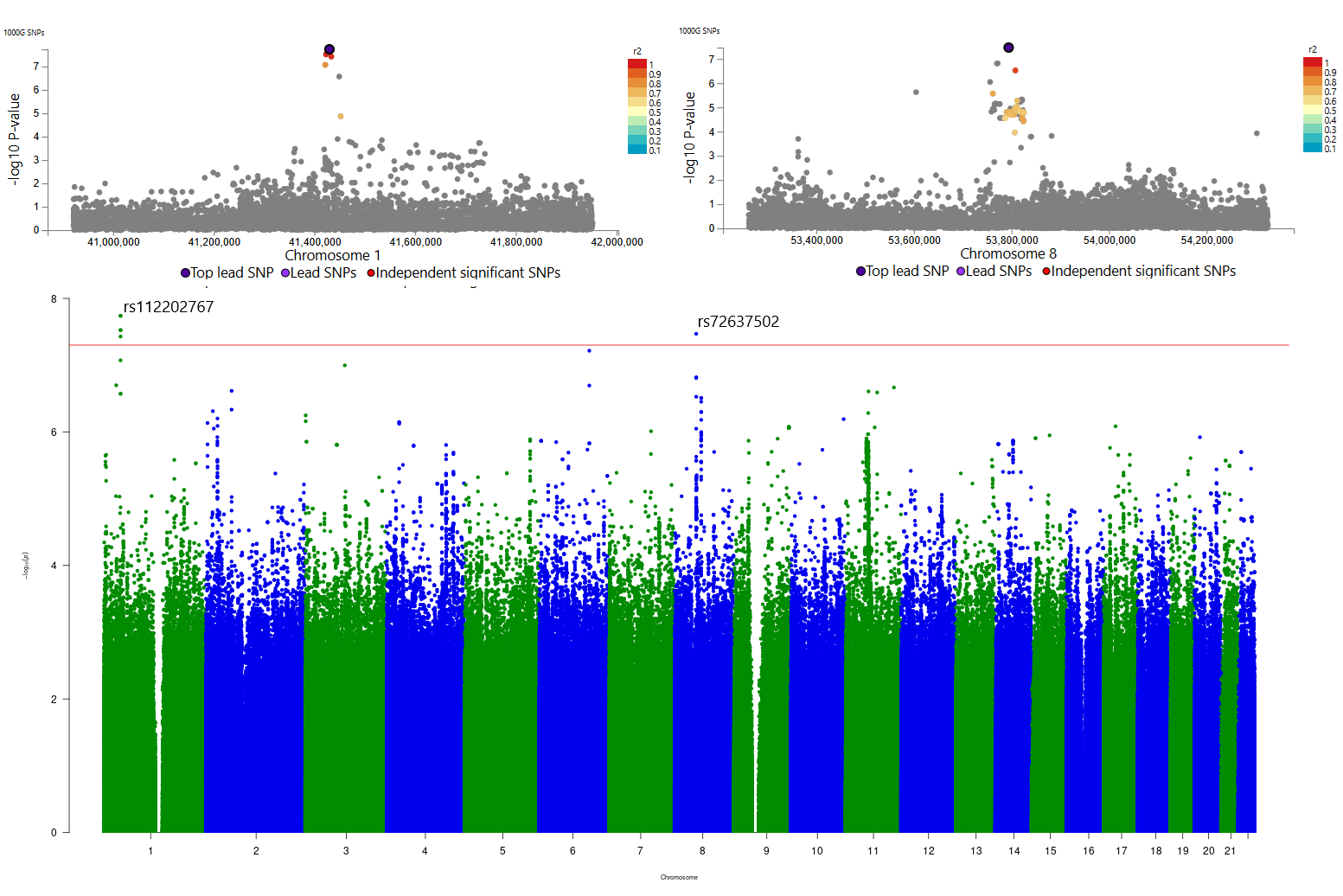


**Figure S6:** Manhattan plot and regional plots of Inhalants Lifetime Use in a cross-ancestry meta-analysis

**
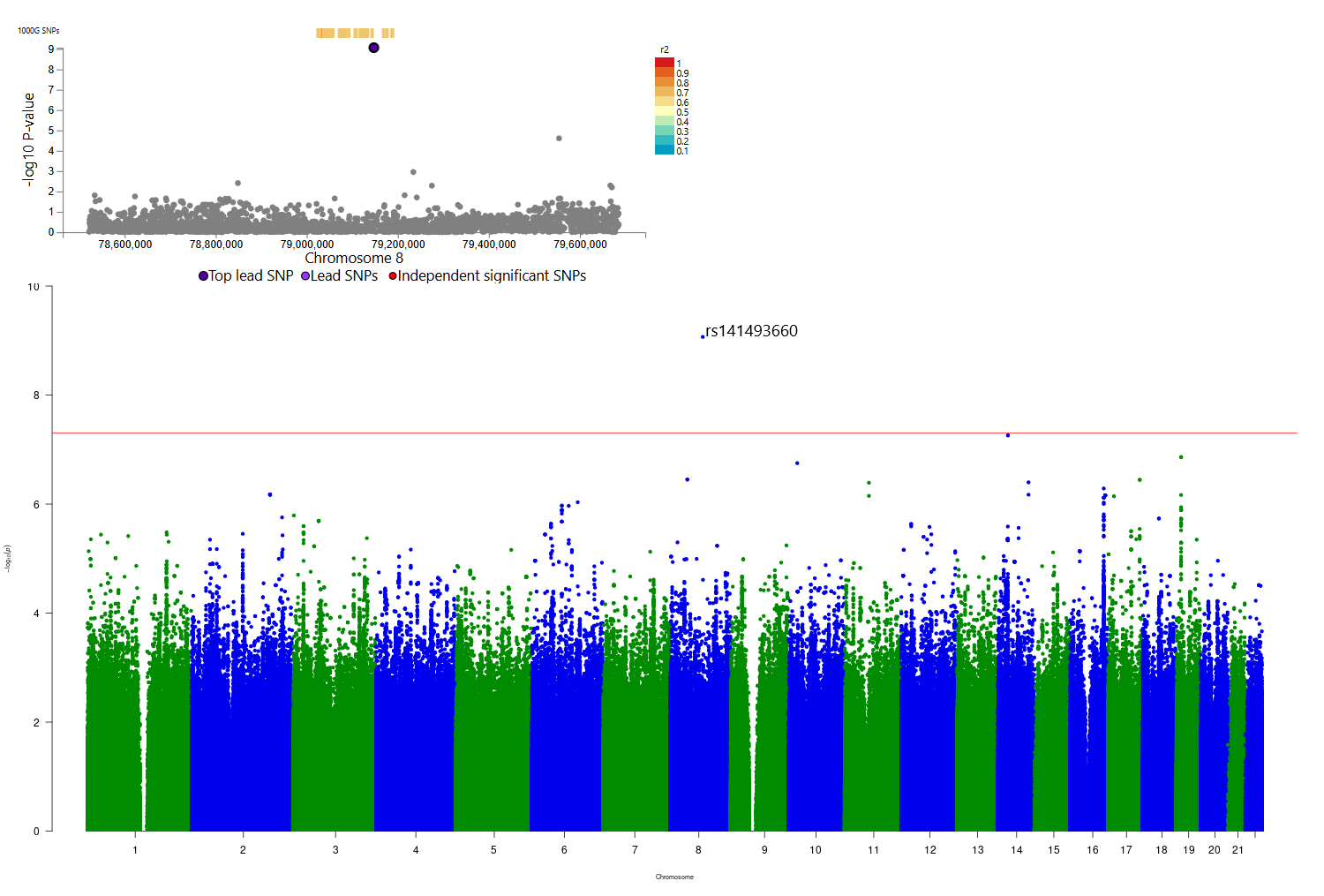
Figure S7:** Manhattan plot and regional plots of Methamphetamine Lifetime Use in AMR

**
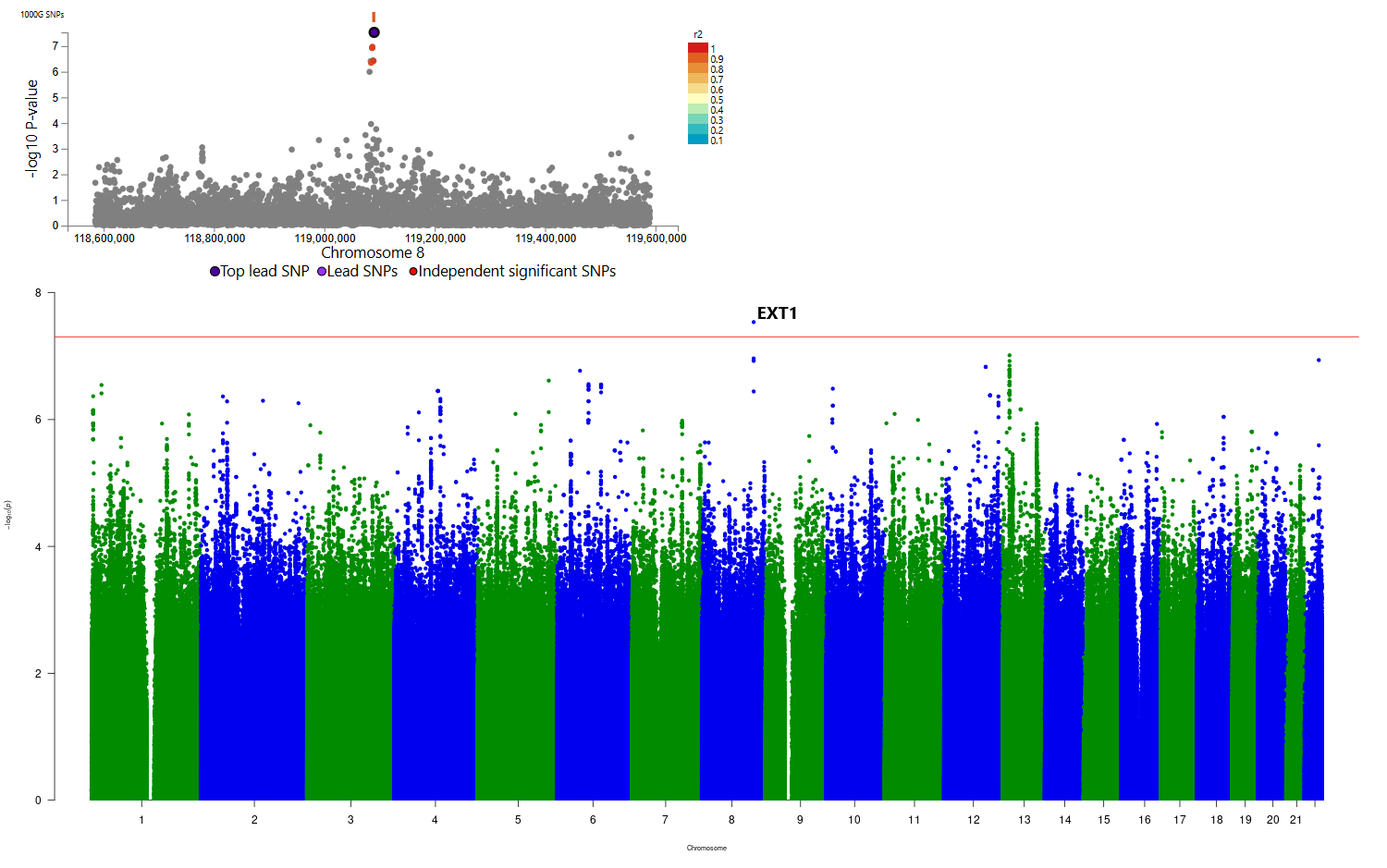
Figure S8:** Manhattan plot and regional plots of Methamphetamine Lifetime Use in across-ancestry meta-analysis

**
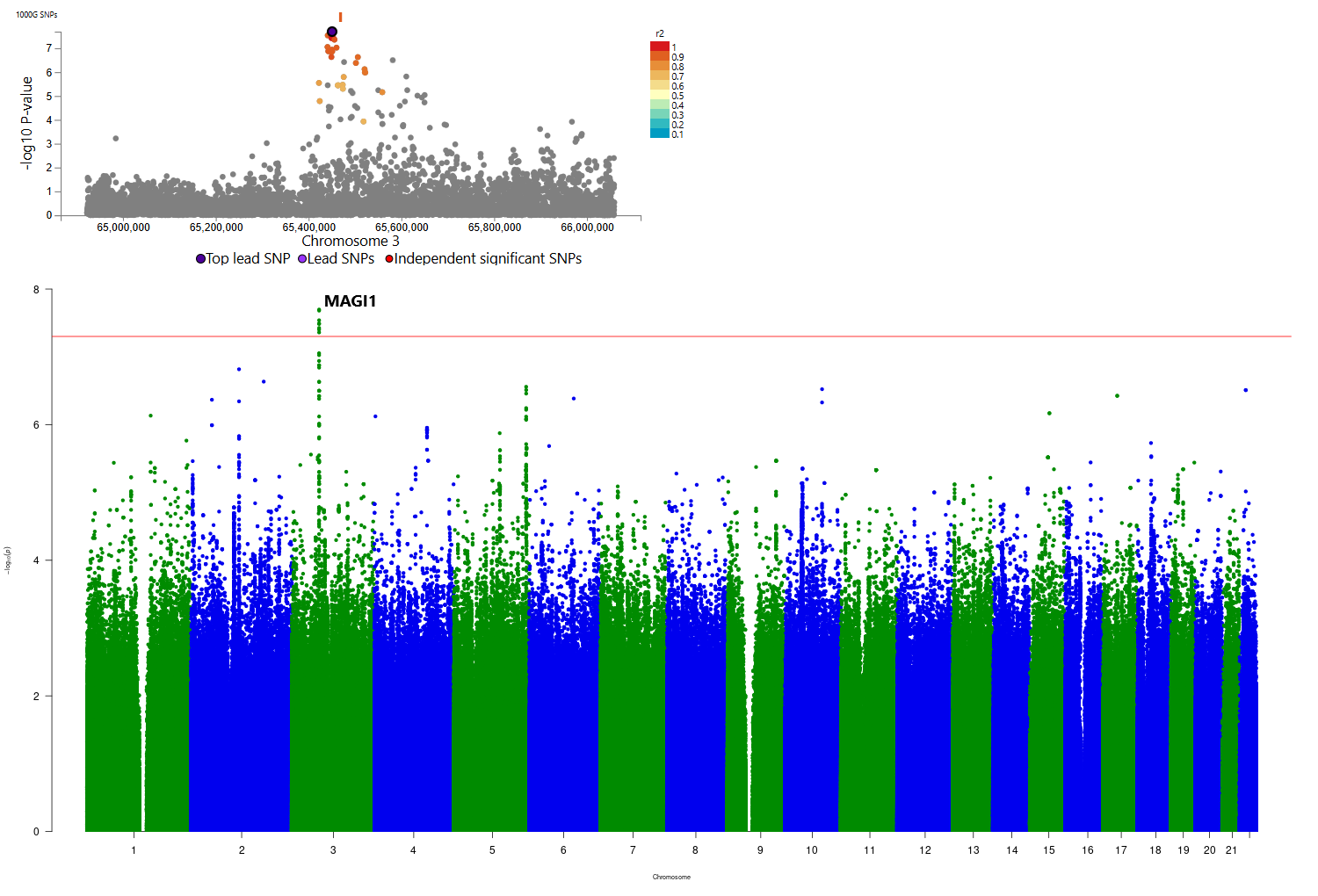
Figure S9:** Manhattan plot and regional plots of Street Opioids Lifetime Use in AFR

**
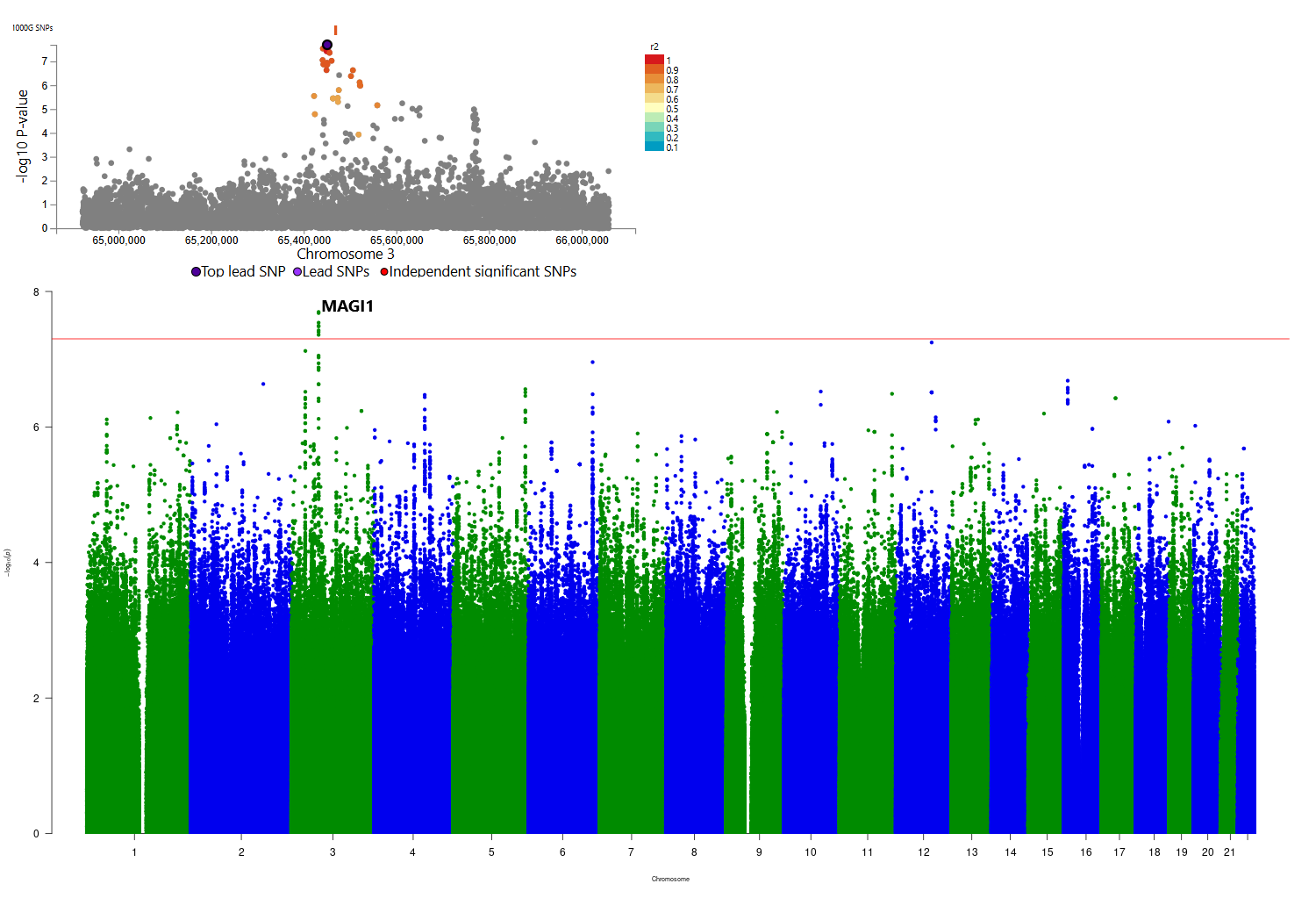
Figure S10:** Manhattan plot and regional plots of Street Opioids Lifetime Use in a cross-ancestry meta-analysis

**
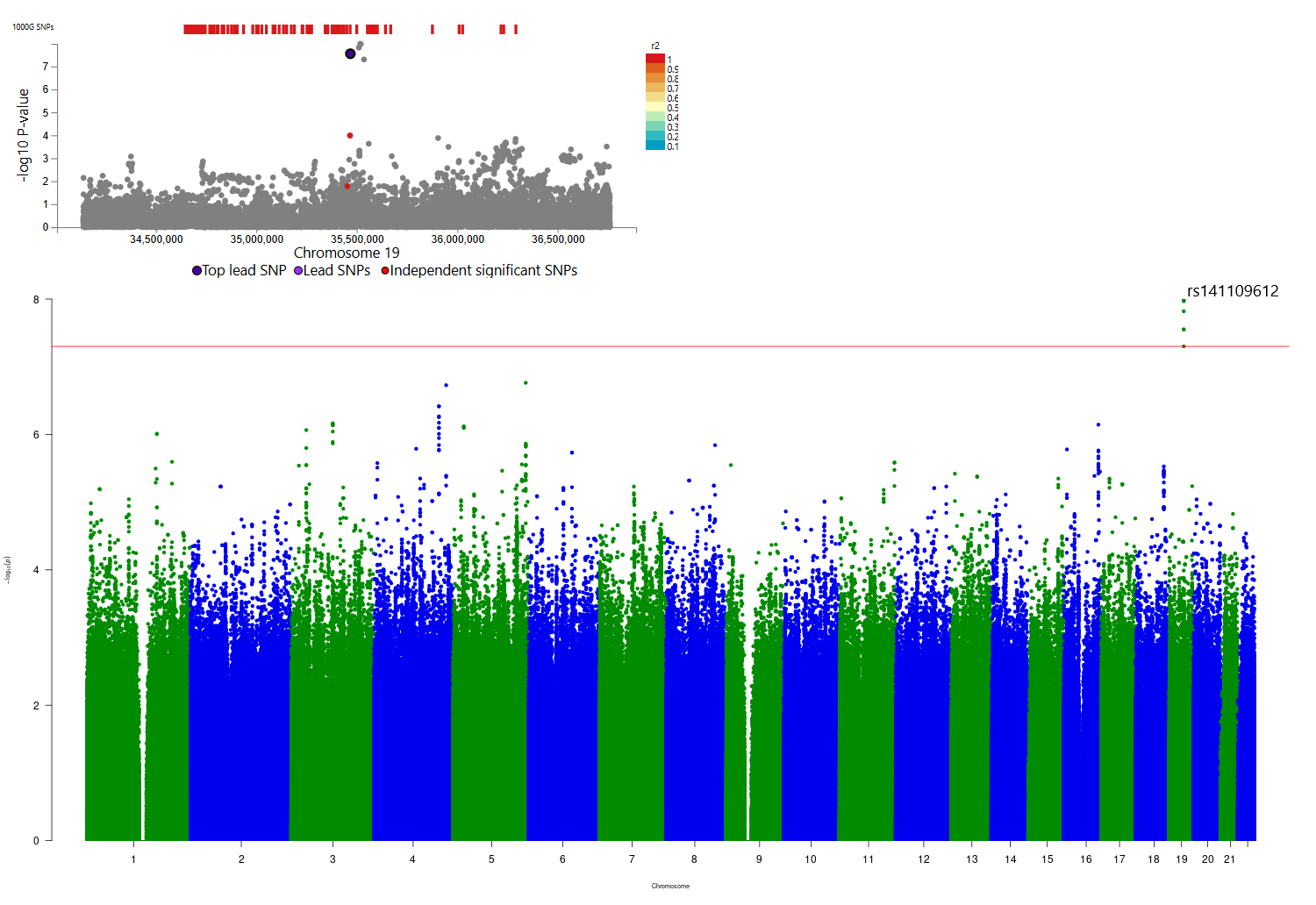
Figure S11:** Manhattan plot and regional plots of Prescription Opioids Lifetime Use in AFR

**
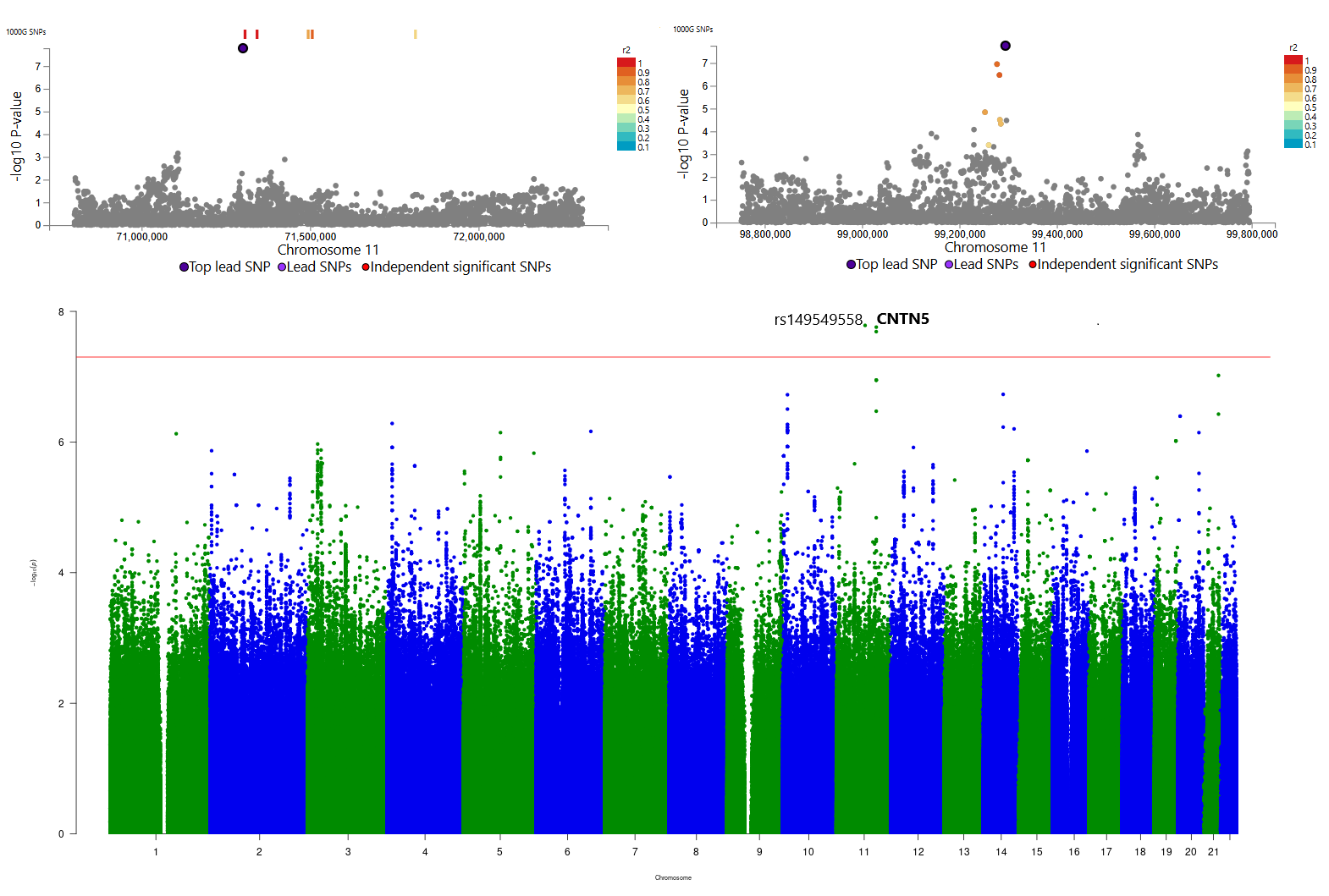
Figure S12:** Manhattan plot and regional plots of Prescription Opioids Lifetime Use in AMR

**
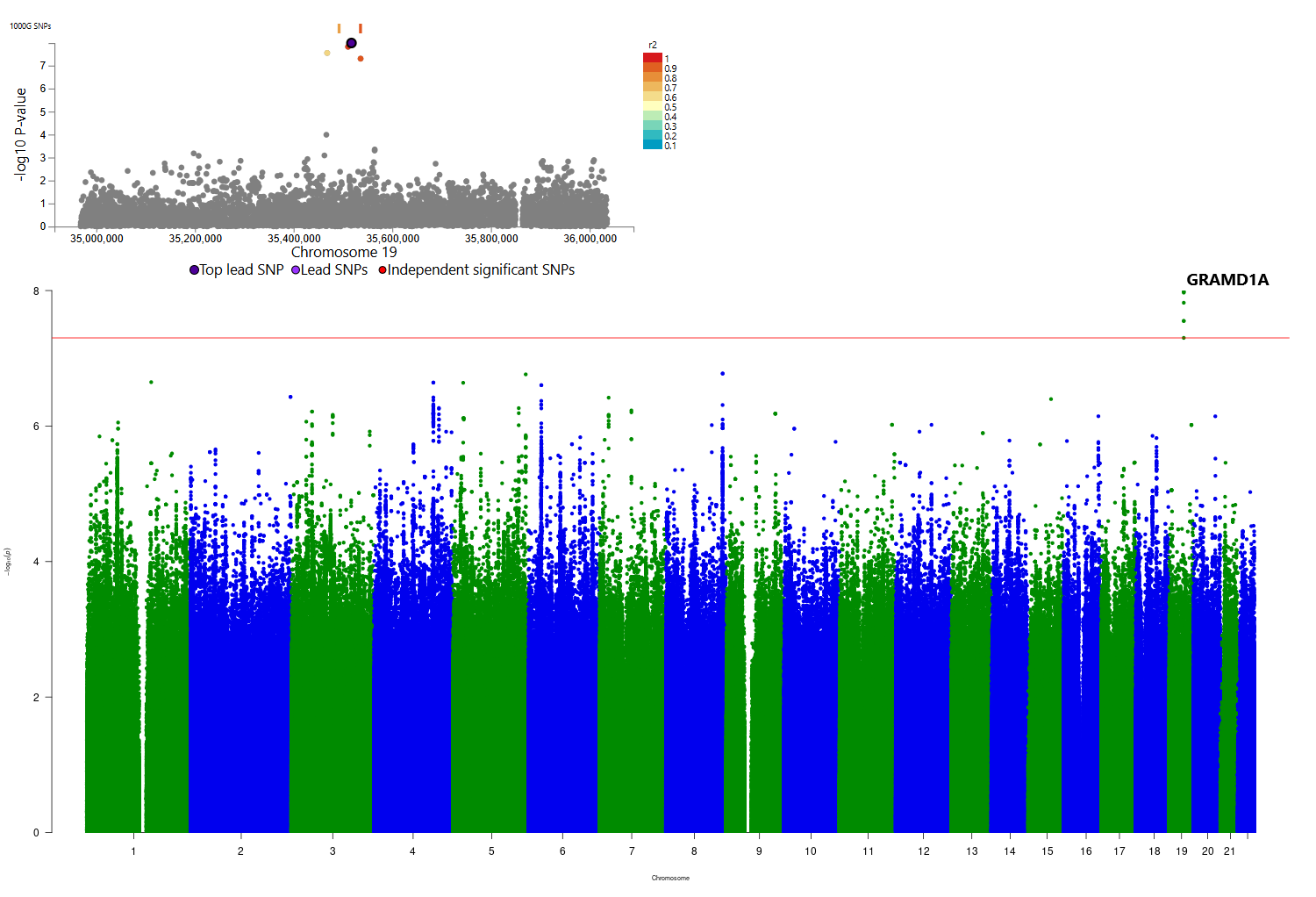
Figure S13:** Manhattan plot and regional plots of Prescription Opioids Lifetime Use in a cross-ancestry meta-analysis

**
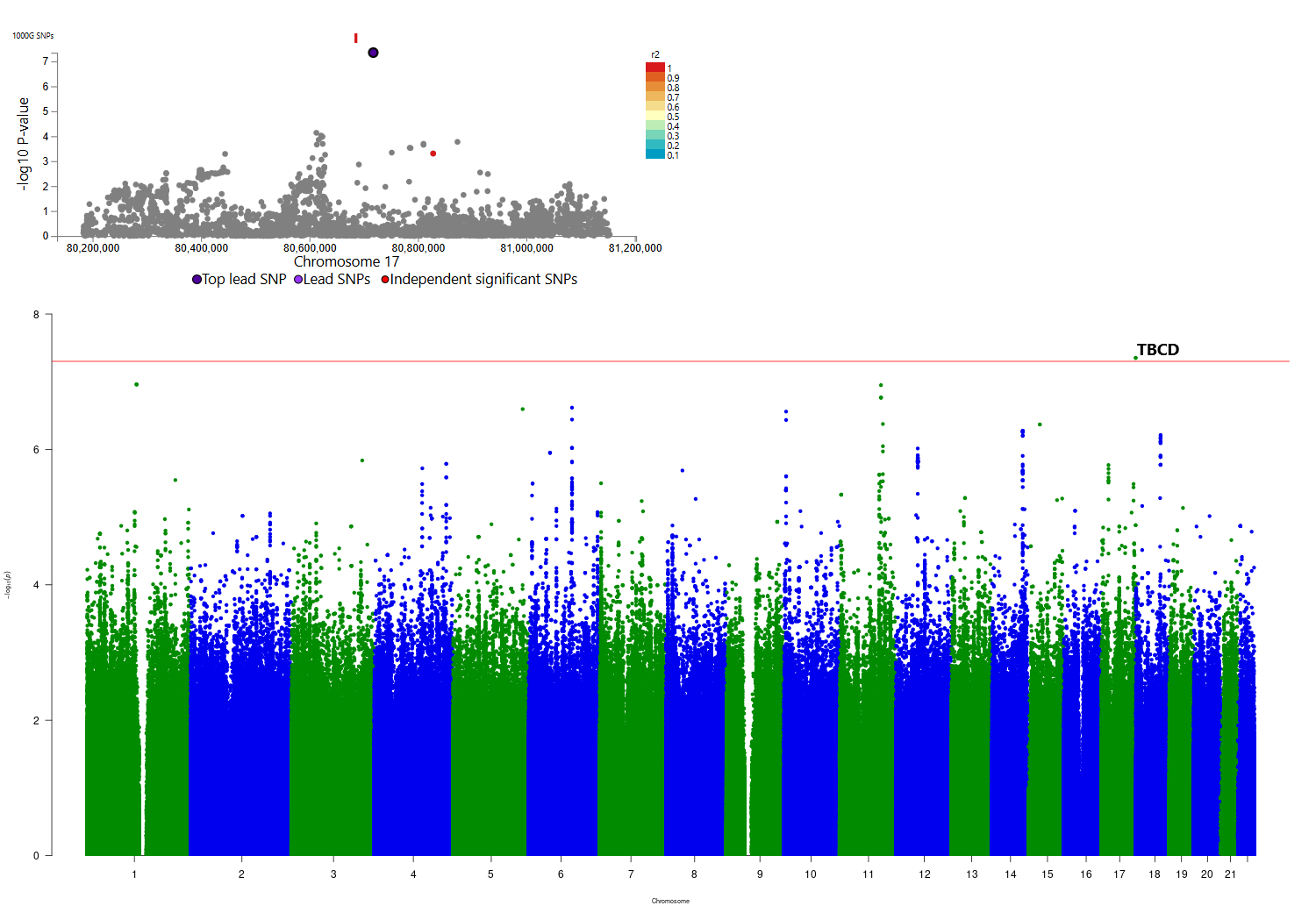
Figure S14:** Manhattan plot and regional plots of Prescription Stimulants Lifetime Use in AMR

**
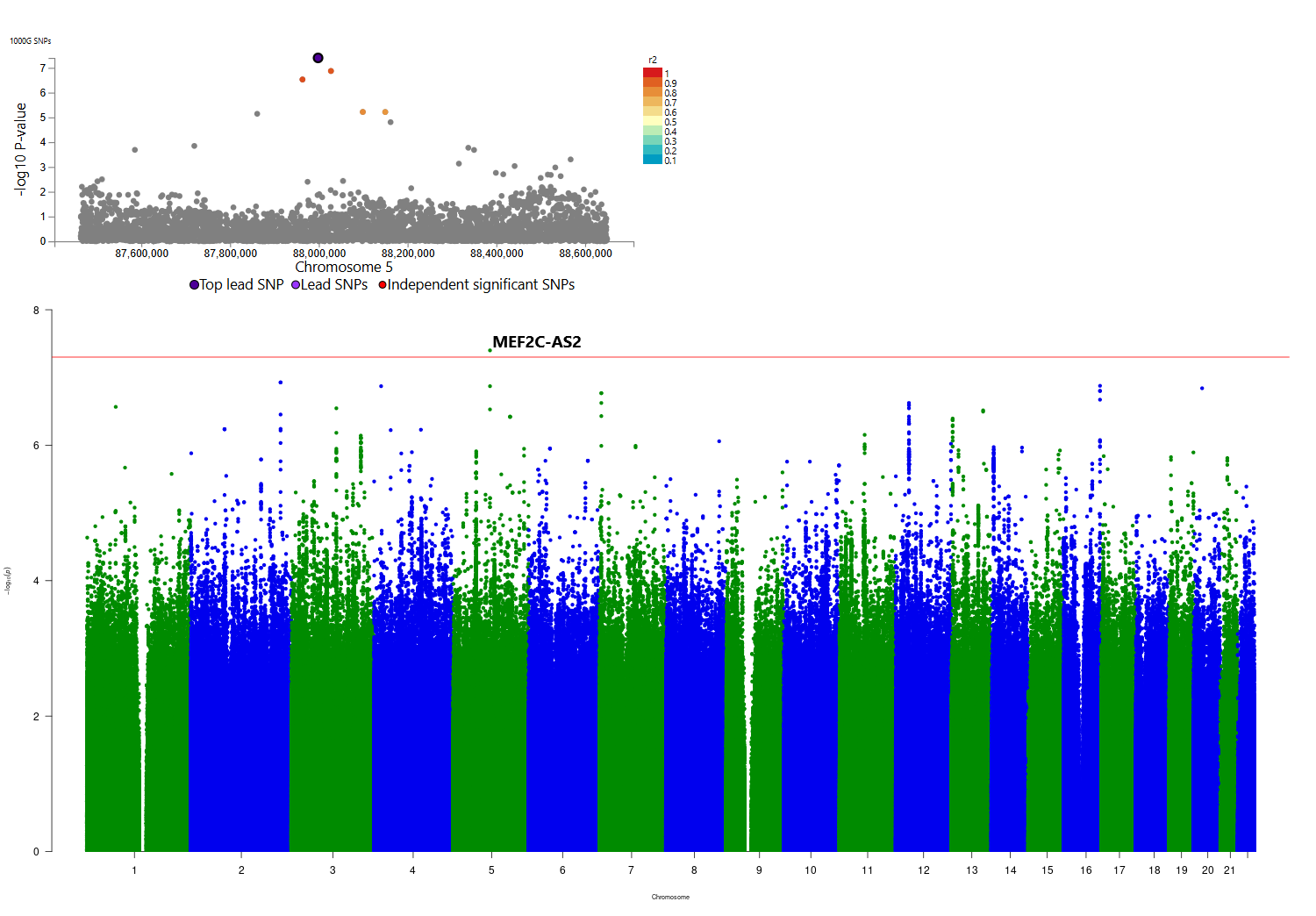
Figure S15:** Manhattan plot and regional plots of Prescription Stimulants Lifetime Use in a cross-ancestry meta-analysis

**
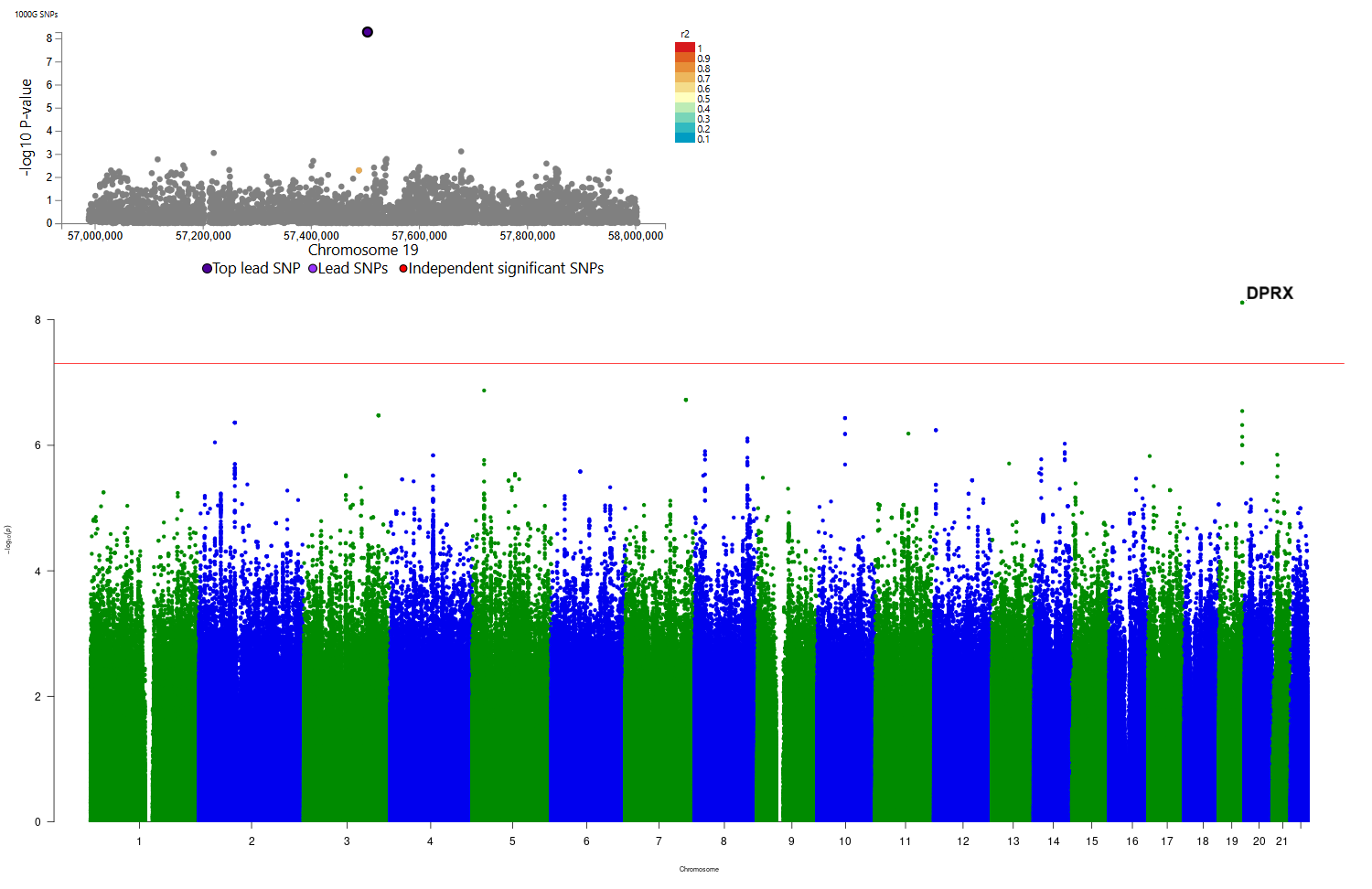
**

**Figure S16:** Manhattan plot and regional plots of Substance Lifetime Use in AFR

**
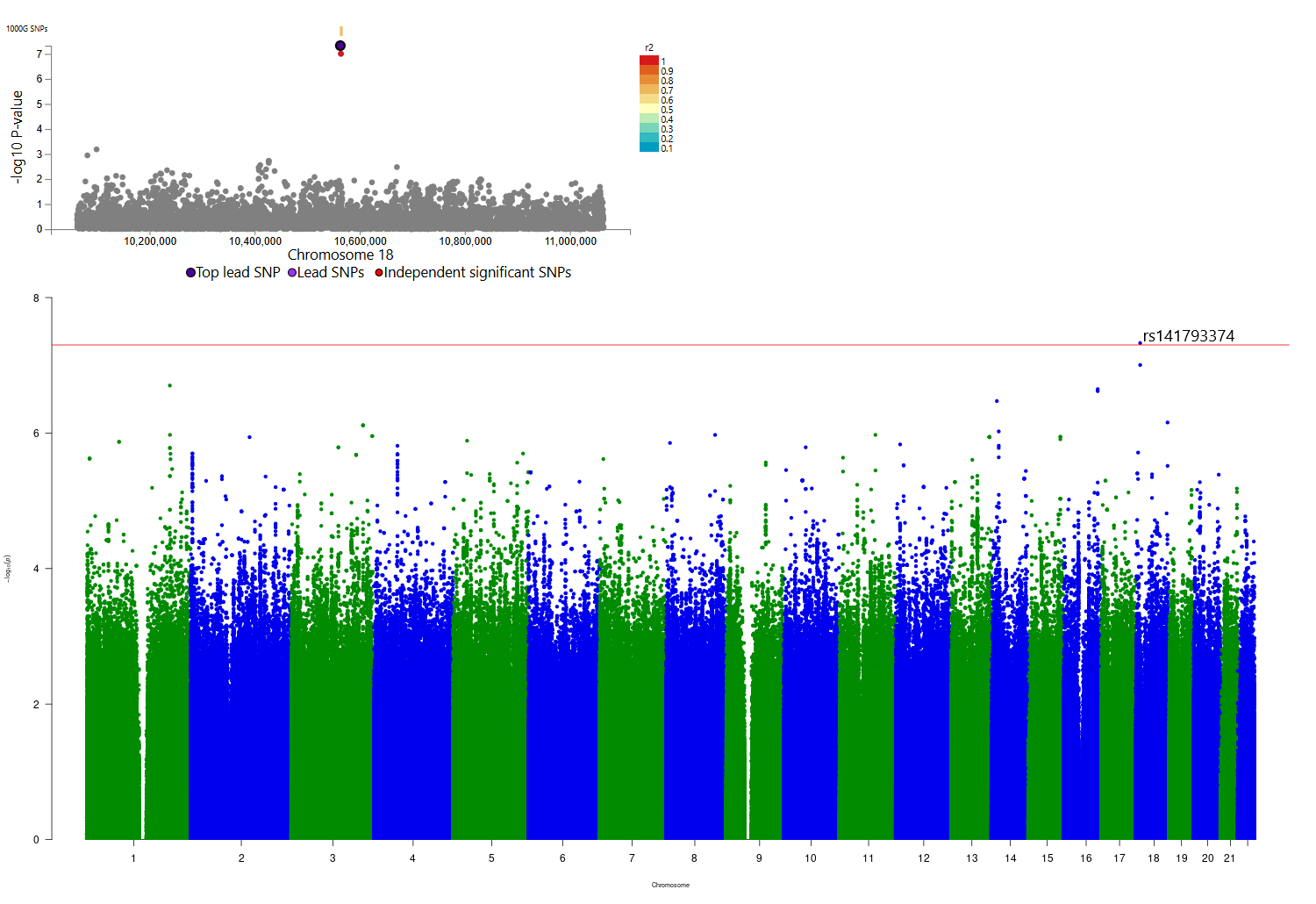
Figure S17:** Manhattan plot and regional plots of Number of Lifetime Substances Used in AFR

**
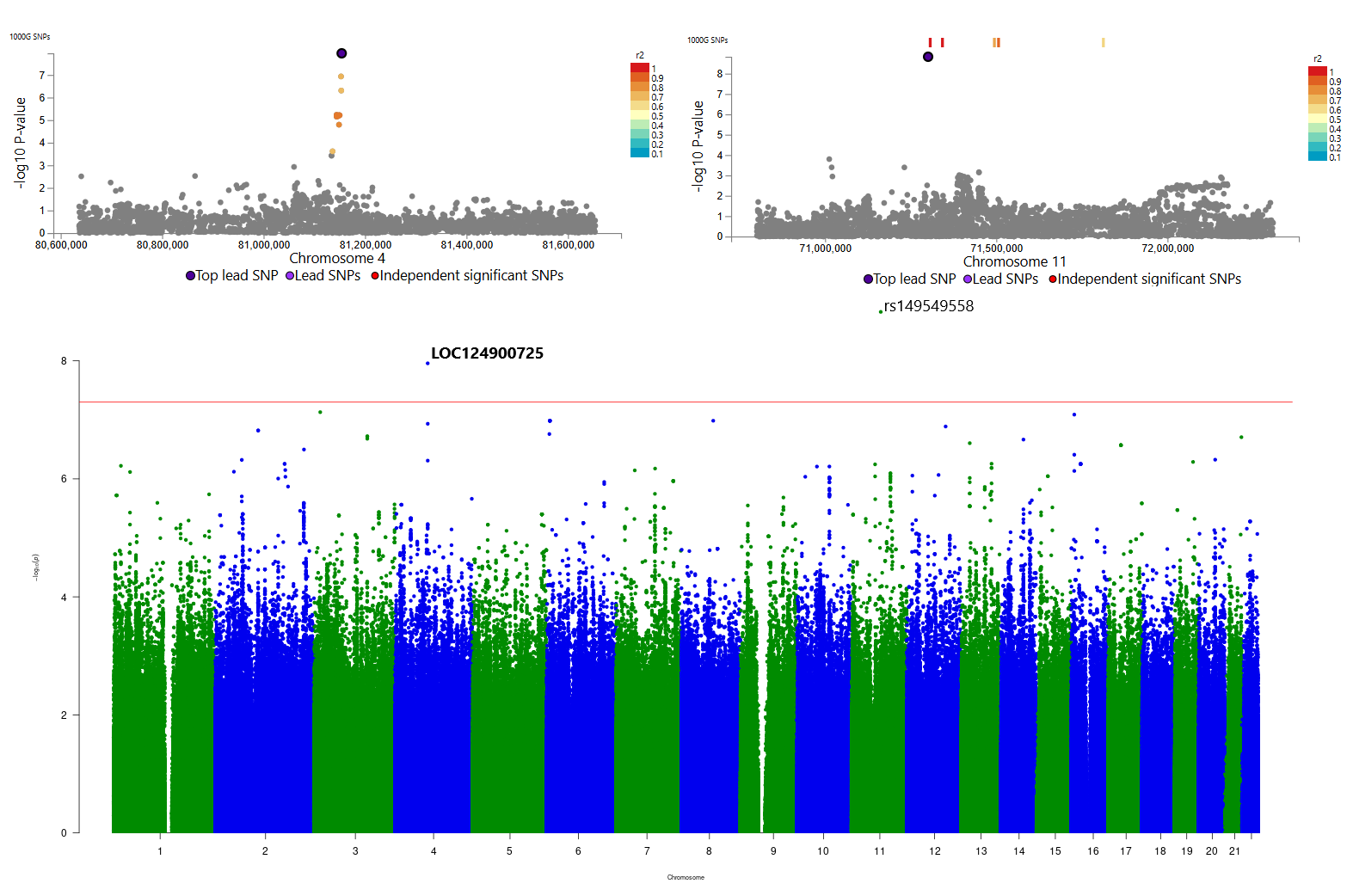
Figure S18:** Manhattan plot and regional plots of Number of Lifetime Substances Used in AMR

**
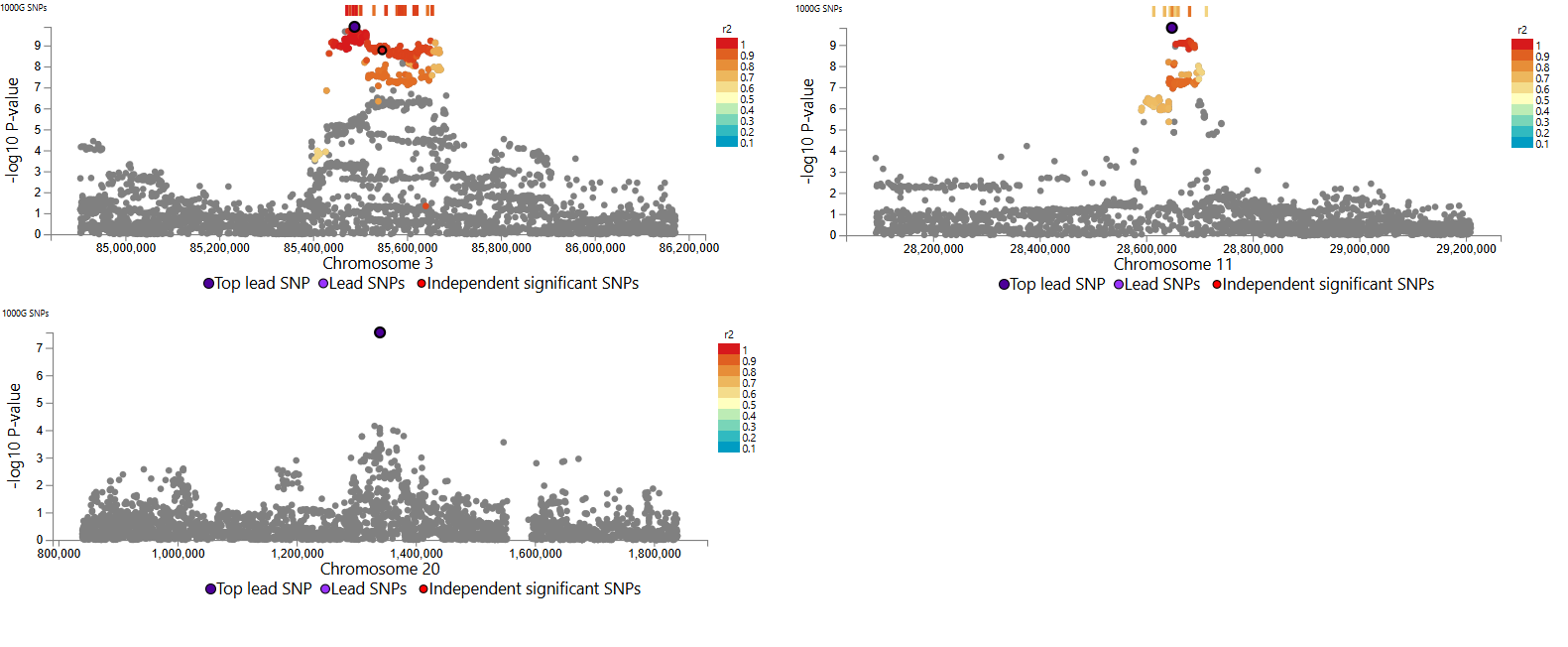
Figure S19:** Regional plots of Substance Lifetime Use in EUR

**
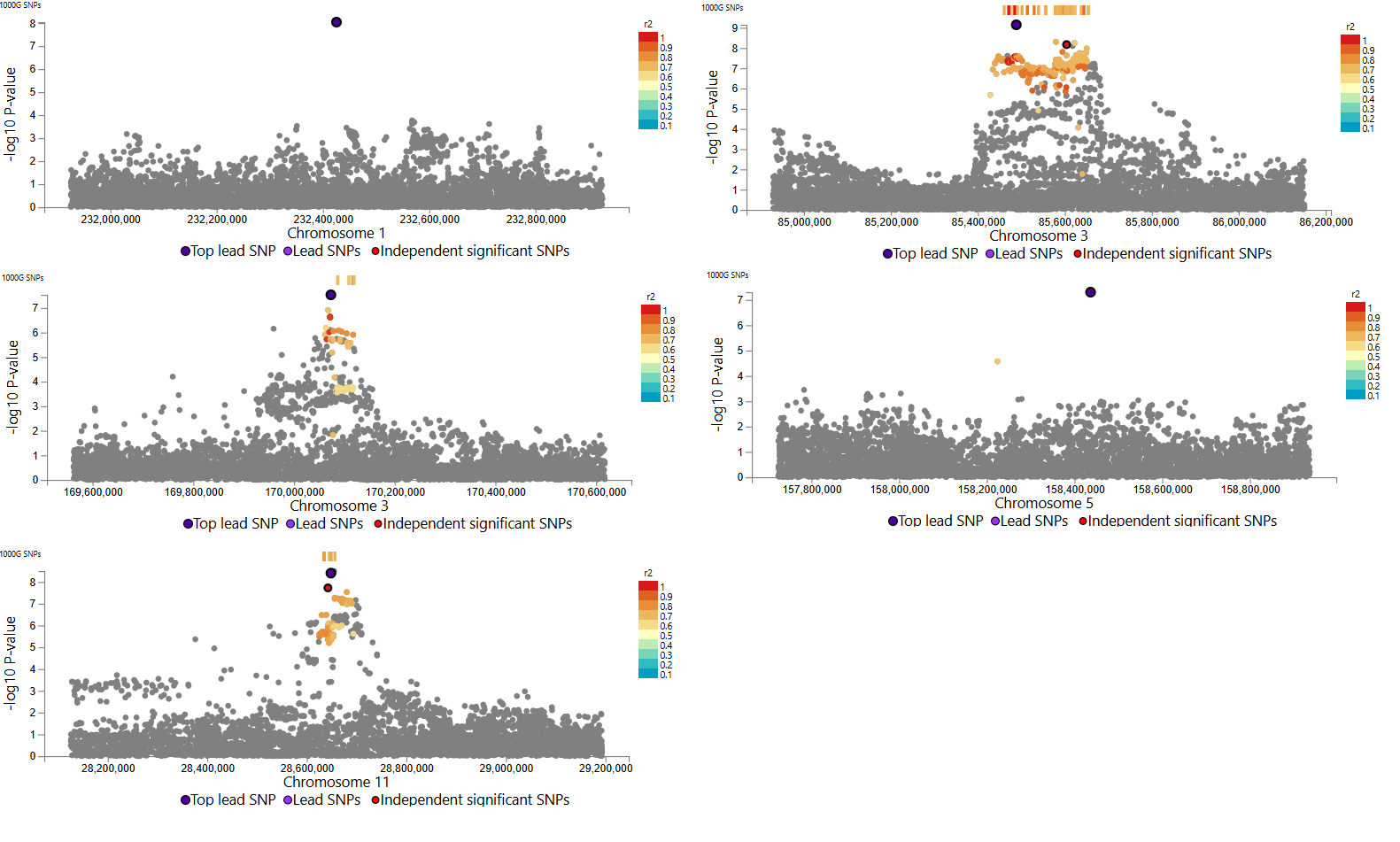
Figure S20:** Regional plots of Substance Lifetime Use in a cross-ancestry meta-analysis

**
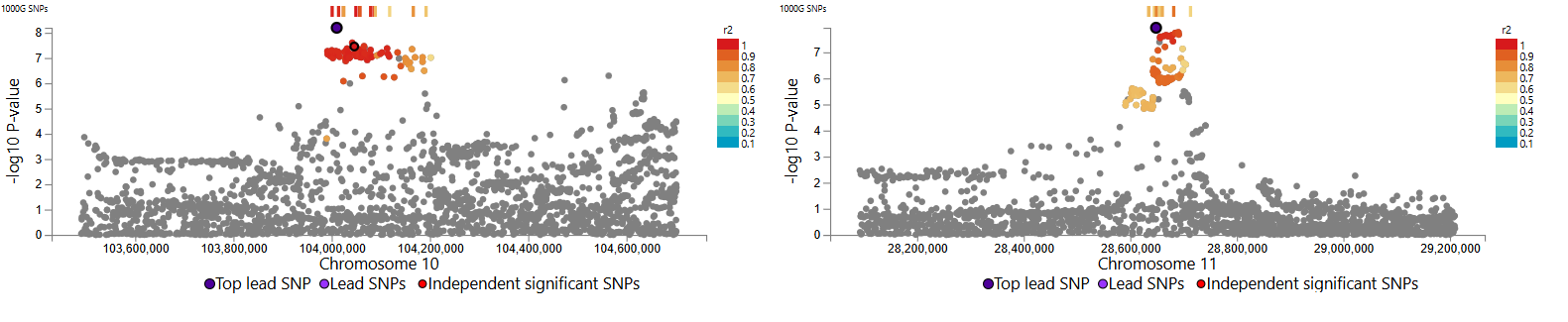
Figure S21:** Regional plots of Number of Lifetime Substances Used in EUR

**
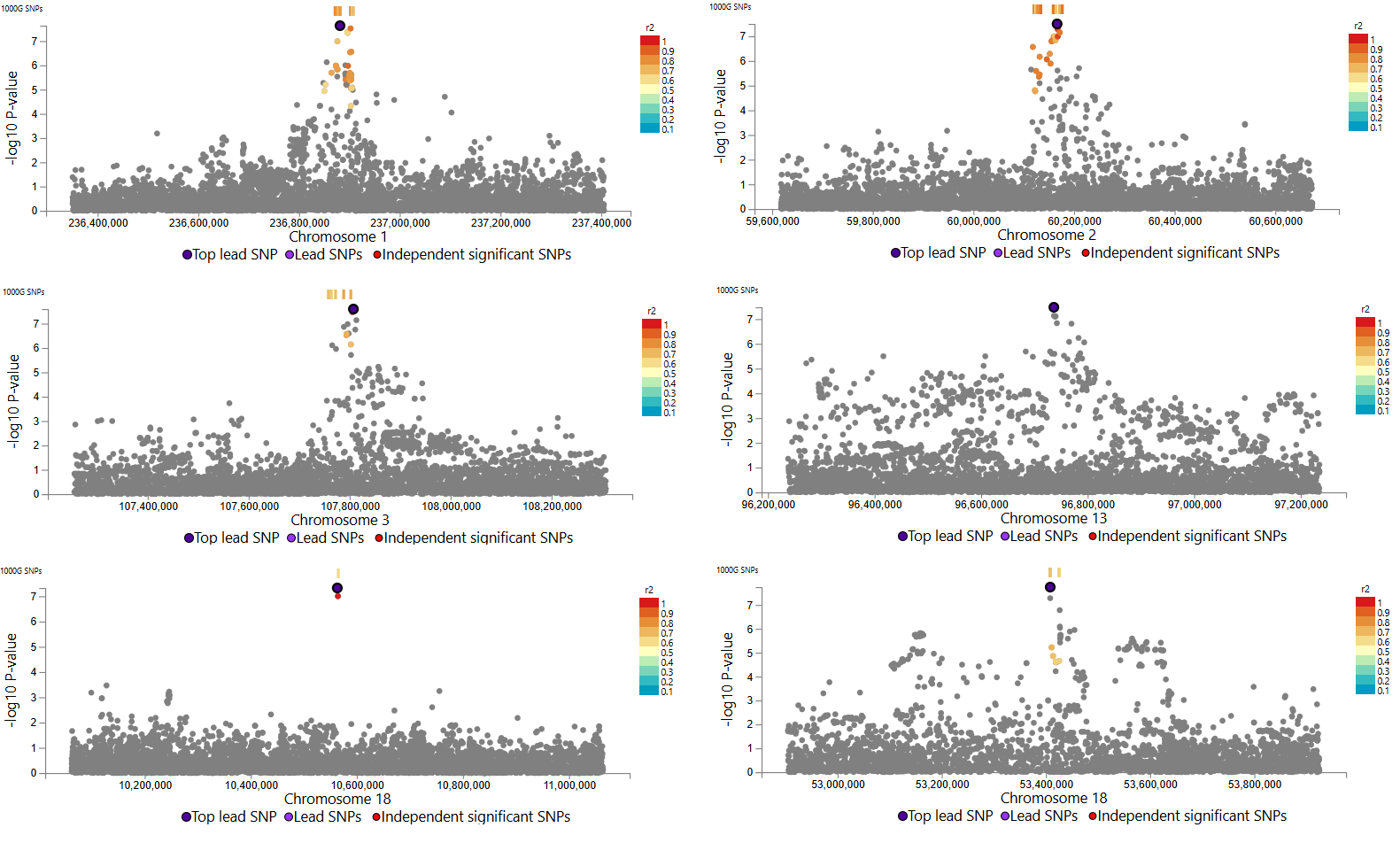
Figure S22:** Regional plots of Number of Lifetime Substances Used in a cross-ancestry meta-analysis


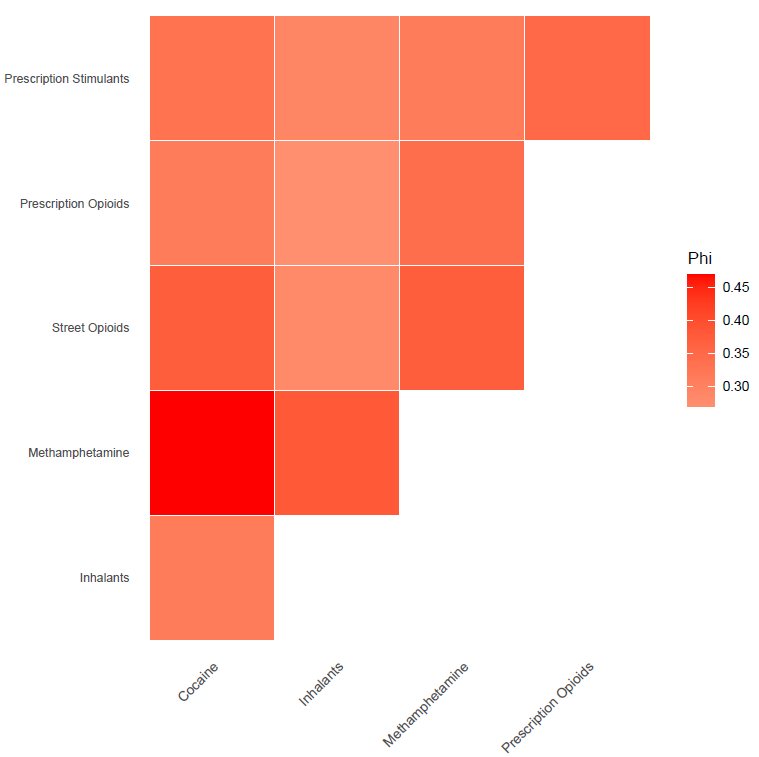


**Figure S23:** Inter-trait phenotypic correlations between all six individual substance Lifetime Use traits in AMR.


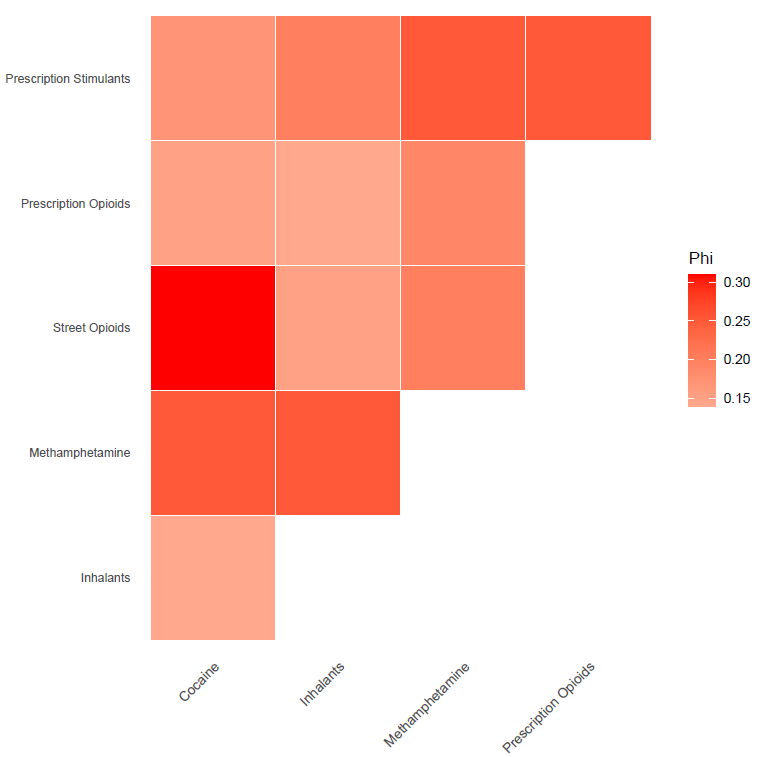


**Figure S24:** Inter-trait phenotypic correlations between all six individual substance Lifetime Use traits in AFR.


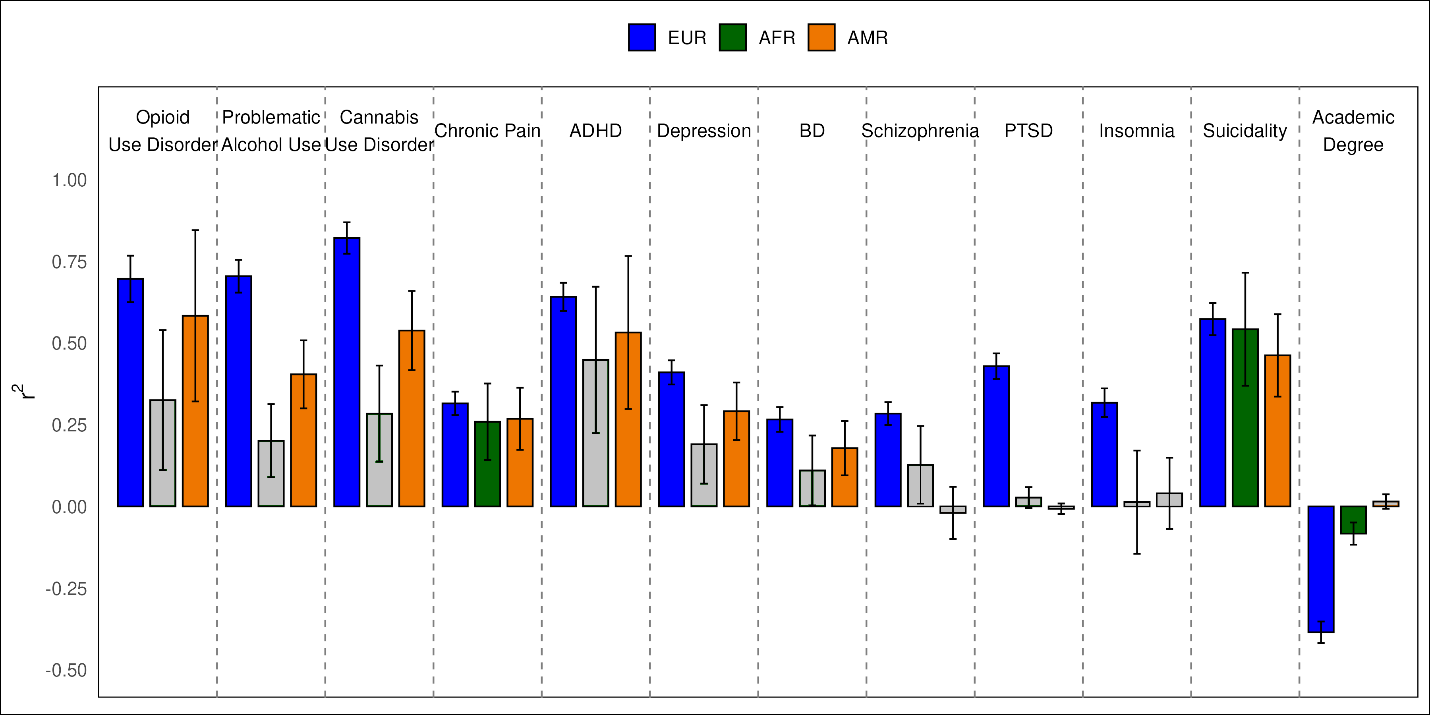


**Figure S25:** Cross-ancestral genetic correlations between SubLU in AMR and AFR and a list of traits of interest in EUR, as calculated by Popcorn. The blue plots show the genetic correlation between these traits in EUR, as calculated by LDSC (figure 3 in the main manuscript), and they are presented here for comparison. Gray plots represent non-significant effects. For every trait, the order of ancestries, from left to right, is EUR, AFR, AMR ADHD: attention-deficit/hyperactivity disorder; BD: bipolar disorder; CanUD: cannabis use disorder; PAU: problematic alcohol use; OUD: opioid use disorder; PTSD: post-traumatic stress disorder].


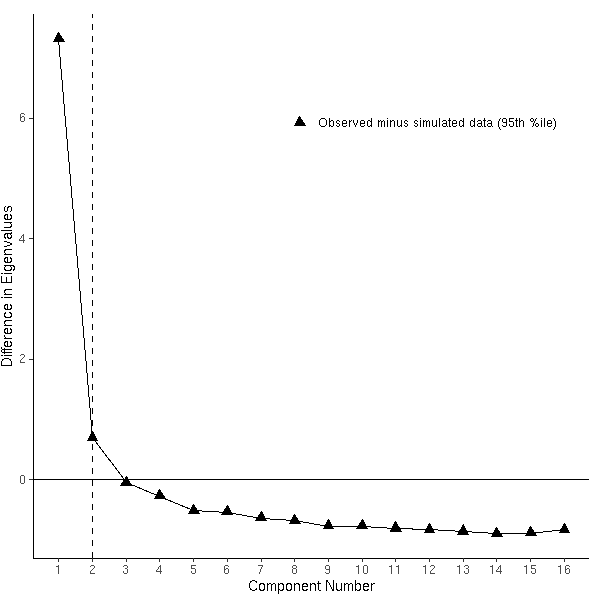


**Figure S26:** gSEM: Parallel analysis results, indicating a two-factor solution. Parallel analysis (paLDSC) results using the standardized correlation matrix of the 15 traits. The psLDSC function of the genomic structural equation modeling package derives the suggested factors to extract (x-axis=Component Number) by comparing eigenvalues determined using the standardized genetic correlation matrix with eigenvalues determined using stimulated null correlation matrix data (y-axis=Difference in Eigenvalues).
