# Supplementary material for: Differences and similarities between the genetic architecture of lifetime substance use across different substances
Source: Psychol Med. 2025 Jul 30;55:e219. doi: 10.1017/S0033291725101293 (PMC12341034; doi:10.1017/S0033291725101293)
Supplement: Bright et al. supplementary material [file S0033291725101293sup001.zip › S0033291725101293sup001/Supplementary Methods.docx]

**Supplementary Methods**

**Cohorts**

We included subjects of European (EUR), African (AFR), and Admixed American (or Latino; AMR) ancestries, from the All of Us (AoU; v7) biobank. Genotyping and quality control procedures were described previously [1]. Lifetime use (LU) of cocaine, methamphetamine, inhalants, street opioids, prescription opioids and prescription stimulants phenotypes were defined using a lifestyle survey; participants whose response for the question “In your lifetime, which of the following substances have you ever used?” included the relevant substance were considered as cases, while all other participants were assigned as controls. The “any substance” (SubLU) phenotype was defined using the same survey, considering as a case any subject whose response included one or more of the substances listed above (i.e., cocaine, methamphetamine, inhalants, street opioids, prescription opioids and prescription stimulants), assigning all the other participants (who did not use any of the aforementioned substances) as controls. A quantitative trait of “number of substances used” (nSubLU) was defined using the same survey as a sum, adapting participants’ count according to the number of substances they listed as “ever used” in their lifetime (a range of 0-6 substances).

**GWAS analysis and meta-analysis**

GWAS analyses were conducted using PLINK 2.0. The six individual substance LU binary traits and the “any substance” (SubLU) trait were analyzed for the three ancestries (EUR, AFR, AMR) using logistic regression, with sex, age, and the first ten PCs of ancestry as covariates. We removed subjects due to relatedness, based on previously published relatedness data [1], with a kinship coefficient cutoff of 0.1. We attempted to remove as few cases as possible – that is, we retained affected rather than unaffected relatives, wherever possible. Variants were excluded due to minor allele frequency (MAF) <0.1% and Hardy-Weinberg equilibrium (HWE) p<1x10^-6^. The quantitative “number of substances used” (nSubLU) was analyzed for the same three ancestries using linear regression with the same covariates. Variants with MAF <0.1% were excluded. For every trait, cross-ancestry meta-analysis was performed using METAL [2] . In all the analyses we applied a standard genome-wide multiple testing correction (p<5x10^-8^). The association results were visualized using the R package qqman [3]. Regional plots were created using FUMA [4].

**Genetic Correlations and SNP-based heritability**

We used linkage disequilibrium score regression (LDSC) [5] based on the linkage disequilibrium (LD) reference from the 1000 Genomes data for all EUR cohorts. We calculated SNP-based heritability (h^2^) for all the traits, including inflation (Lamda), the intercept and the attenuation ratio, and inter-trait genetic correlations between all six individual substance lifetime use traits. After Bonferroni correction for 15 tests, the statistical significance threshold was set at p=0.0033. Then, based on publicly-available large-scale summary statistics [6-15] (**Table S1**), we calculated the genetic correlation between each trait, including the composite traits SubLU and nSubLU, and eleven selected traits. These related to substance dependence or use disorder, common psychiatric illnesses, and traits that are commonly associated with the use of the substances (e.g., ADHD, which is associated with stimulant use [16], and chronic pain, which is associated with opioid use [17]). For all traits we used the effective sample size, and h² was converted to the liability scale using the sample prevalence and population prevalence, based on existing data [18-29]. After Bonferroni correction for 88 tests, the statistical significance threshold was set on p=0.0006.

**Phenotypic Correlations**

We estimated inter-trait phenotypic correlation using chi-square and phi coefficient (r_φ_).

**Local Genetic Correlations**

We used local analysis of covariant association (LAVA) [30] to calculate inter-trait local genetic correlations between all six substance lifetime use traits. The genome was divided into 2,495 genomic regions, to attain minimum LD between the regions and maintain an approximate equal size of around 1 MB. Breakpoints between the regions were computed according to the LD between neighboring SNPs as described in ref. [30], to maintain the regions as relatively independent. Univariate local correlations were conducted for each trait. For every trait, only regions that reached the significance threshold of p<0.05 were used to calculate genetic correlations with the other traits. Local genetic correlations were calculated for all pairs (a total of 15 pairs). Then, based on publicly-available large-scale summary statistics (**Table S1**), we calculated the local genetic correlations between each trait – including SubLU and nSubLU – and eleven selected traits of interest using the same procedure as described above.

**Cross-Ancestry Genetic Correlations**

We used Popcorn [31] to calculate the cross-ancestry genetic correlations between the SubLU trait (but not other substance use traits including individual traits, due to low power) in AFR and AMR populations and a selected list of traits in EUR. We used the same set of traits that were measured for genetic correlation among EUR using LDSC (see above). We used the 1000 Genomes data as reference [32]. We applied the Benjamini–Hochberg procedure for correction of false discovery rate (FDR).

**Genomic Structural Equation Modeling (gSEM)**

We utilized genomic structural equation modeling (gSEM) [33] to examine the latent factor structure underlying the six individual substance LU traits, and nine other psychiatric and health-related traits, which are associated with substance use, and with these substances specifically. First, we evaluated the standardized genetic correlation matrix via heatmap/dendrogram and parallel analyses (paLDSC, in the gSEM package [34]) to guide factor selection. Second, based on the suggested factors from the first step, we evaluated two- and three-factor model solutions via exploratory factor analyses (EFAs). To be considered adequately loading onto a factor, the loading had to be >0.30 and we also allowed for cross-loading if the loading was >0.30 for multiple factors. Finally, we performed a confirmatory factor analysis (CFA) and assessed model fit via the following conventions: comparative fit index (CFI) ≥ 0.90 indicates adequate fit and CFI ≥ 0.95 excellent fit while standardized root mean square residual (SRMR) ≤ 0.08 suggests good fit.

**References (Supplementary Methods)**

1. Bick, A.G., et al., *Genomic data in the All of Us Research Program.* Nature, 2024. **627**(8003).

2. Willer, C.J., Y. Li, and G.R. Abecasis, *METAL: fast and efficient meta-analysis of genomewide association scans.* Bioinformatics, 2010. **26**(17): p. 2190-2191.

3. Turner, S., *qqman: an R package for visualizing GWAS results using Q-Q.* The Journal of Open Source Software, 2018.

4. Watanabe, K., et al., *Functional mapping and annotation of genetic associations with FUMA.* Nat Commun, 2017. **8**(1): p. 1826.

5. Bulik-Sullivan, B.K., et al., *LD Score regression distinguishes confounding from polygenicity in genome-wide association studies.* Nat Genet, 2015. **47**(3): p. 291-5.

6. Deak, J.D., et al., *Genome-wide association study in individuals of European and African ancestry and multi-trait analysis of opioid use disorder identifies 19 independent genome-wide significant risk loci.* Mol Psychiatry, 2022. **27**(10): p. 3970-3979.

7. Zhou, H., et al., *Multi-ancestry study of the genetics of problematic alcohol use in over 1 million individuals.* Nat Med, 2023. **29**(12): p. 3184-3192.

8. Levey, D.F., et al., *Multi-ancestry genome-wide association study of cannabis use disorder yields insight into disease biology and public health implications.* Nature Genetics, 2023. **55**(12): p. 2094-2103.

9. Johnston, K.J.A., et al., *Genome-wide association study of multisite chronic pain in UK Biobank.* PLoS Genet, 2019. **15**(6): p. e1008164.

10. Demontis, D., et al., *Genome-wide analyses of ADHD identify 27 risk loci, refine the genetic architecture and implicate several cognitive domains.* Nat Genet, 2023. **55**(2): p. 198-208.

11. Trubetskoy, V., et al., *Mapping genomic loci implicates genes and synaptic biology in schizophrenia.* Nature, 2022. **604**(7906): p. 502-508.

12. Nievergelt, C.M., et al., *Genome-wide association analyses identify 95 risk loci and provide insights into the neurobiology of post-traumatic stress disorder.* Nat Genet, 2024. **56**(5): p. 792-808.

13. Watanabe, K., et al., *Genome-wide meta-analysis of insomnia prioritizes genes associated with metabolic and psychiatric pathways.* Nat Genet, 2022. **54**(8): p. 1125-1132.

14. Docherty, A.R., et al., *GWAS Meta-Analysis of Suicide Attempt: Identification of 12 Genome-Wide Significant Loci and Implication of Genetic Risks for Specific Health Factors.* Am J Psychiatry, 2023. **180**(10): p. 723-738.

15. Levey, D.F., et al., *Bi-ancestral depression GWAS in the Million Veteran Program and meta-analysis in >1.2 million individuals highlight new therapeutic directions.* Nat Neurosci, 2021. **24**(7): p. 954-963.

16. Wilens, T.E., et al., *Misuse and diversion of stimulants prescribed for ADHD: A systematic review of the literature.* Journal of the American Academy of Child and Adolescent Psychiatry, 2008. **47**(1): p. 21-31.

17. Weiss, R.D., et al., *Reasons for opioid use among patients with dependence on prescription opioids: the role of chronic pain.* J Subst Abuse Treat, 2014. **47**(2): p. 140-5.

18. Martins, S.S., et al., *Changes in US Lifetime Heroin Use and Heroin Use Disorder: Prevalence From the 2001-2002 to 2012-2013 National Epidemiologic Survey on Alcohol and Related Conditions.* JAMA Psychiatry, 2017. **74**(5): p. 445-455.

19. Han, B., et al., *Prescription Opioid Use, Misuse, and Use Disorders in U.S. Adults: 2015 National Survey on Drug Use and Health.* Ann Intern Med, 2017. **167**(5): p. 293-301.

20. McCabe, S.E. and B.T. West, *Medical and nonmedical use of prescription stimulants: results from a national multicohort study.* J Am Acad Child Adolesc Psychiatry, 2013. **52**(12): p. 1272-80.

21. Barocas, J.A., et al., *Estimated Prevalence of Opioid Use Disorder in Massachusetts, 2011-2015: A Capture-Recapture Analysis.* Am J Public Health, 2018. **108**(12): p. 1675-1681.

22. Grant, B.F., et al., *Prevalence of 12-Month Alcohol Use, High-Risk Drinking, and DSM-IV Alcohol Use Disorder in the United States, 2001-2002 to 2012-2013: Results From the National Epidemiologic Survey on Alcohol and Related Conditions.* JAMA Psychiatry, 2017. **74**(9): p. 911-923.

23. Boness, C.L., et al., *Prevalence of Cannabis Use Disorder: A Meta-Analysis of Population Surveys.* J Stud Alcohol Drugs, 2025. **86**(1): p. 25-38.

24. Yong, R.J., P.M. Mullins, and N. Bhattacharyya, *Prevalence of chronic pain among adults in the United States.* Pain, 2022. **163**(2): p. e328-e332.

25. Polanczyk, G., et al., *The worldwide prevalence of ADHD: a systematic review and metaregression analysis.* Am J Psychiatry, 2007. **164**(6): p. 942-8.

26. Shorey, S., E.D. Ng, and C.H.J. Wong, *Global prevalence of depression and elevated depressive symptoms among adolescents: A systematic review and meta-analysis.* Br J Clin Psychol, 2022. **61**(2): p. 287-305.

27. Castillejos, M.C., et al., *Prevalence of Suicidality in the European General Population: A Systematic Review and Meta-Analysis.* Arch Suicide Res, 2021. **25**(4): p. 810-828.

28. SAMHSA, *Substance Abuse and Mental Gealth Services Administration (SAMHSA): Results from the 2023 National Survey on Drug Use and Health: Detailed Tables*. 2023.

29. WHO, *World Health Organizarion (WHO: Schizophrenia)*. 2022.

30. Werme, J., et al., *An integrated framework for local genetic correlation analysis.* Nature Genetics, 2022. **54**(3): p. 274-+.

31. Brown, B.C., et al., *Transethnic Genetic-Correlation Estimates from Summary Statistics.* American Journal of Human Genetics, 2016. **99**(1): p. 76-88.

32. Genomes Project, C., et al., *A global reference for human genetic variation.* Nature, 2015. **526**(7571): p. 68-74.

33. Grotzinger, A.D., et al., *Genomic structural equation modelling provides insights into the multivariate genetic architecture of complex traits.* Nat Hum Behav, 2019. **3**(5): p. 513-525.

34. Furtjes, A.E., et al., *General dimensions of human brain morphometry inferred from genome-wide association data.* Hum Brain Mapp, 2023. **44**(8): p. 3311-3323.
